# Australian healthcare workers’ perspectives, practices, and perceived barriers to a more environmentally sustainable health system: a cross-sectional survey

**DOI:** 10.1186/s12913-026-14464-8

**Published:** 2026-03-31

**Authors:** Michael J. Loftus, Zerina Lokmic-Tomkins, Owen Eades, Mohammad Asghari-Jafarabadi, Karen Walker-Bone, Adrian Chee, Jess Davies, Ben Dunne, Xenon Ellis, Mike Forrester, Madeleine Gemmell, Michael W. Hii, Ana Hutchinson, Karen M. Kiang, Kylie McIntyre, Lynden J. Roberts, Philip L. Russo, Asma Sohail, Sarah L. McGuinness, Karin Leder

**Affiliations:** 1 Health and Climate Initiative, Faculty of Medicine, Nursing and Health Sciences, Monash University, Melbourne, VIC Australia; 2 Division of Planetary Health, School of Population Health and Preventive Medicine, Monash University, Melbourne, VIC Australia; 3 Department of Infectious Diseases, Alfred Health, Melbourne, VIC Australia; 4 Department of General Medicine, Mildura Base Public Hospital, Mildura, VIC Australia; 5 Victorian Heart Institute, Monash University, Clayton, VIC Australia; 6 School of Nursing and Midwifery, Monash University, Clayton, VIC Australia; 7 School of Translational Medicine, Monash University, Melbourne, VIC Australia; 8 Biostatistics Unit, School of Public Health and Preventive Medicine, Monash University, Melbourne, VIC Australia; 9 Department of Psychiatry, School of Clinical Sciences, Monash University, Clayton, VIC Australia; 10 Cabrini Research, Cabrini Health, Malvern, VIC Australia; 11 Department of Anaesthesia, Austin Health, Heidelberg, VIC Australia; 12 Department of Clinical Care, Melbourne Medical School, University of Melbourne, Melbourne, VIC Australia; 13 Department of Cardiothoracic Surgery, Royal Melbourne Hospital, Parkville, VIC Australia; 14 Strategy, Transformation and Projects, Monash Medical Centre Clayton, Clayton, VIC Australia; 15 Department of Children’s Services, University Hospital Geelong, Geelong, VIC Australia; 16 Sustainable Health Network, Institute for Health Transformation, Deakin University, Geelong, VIC Australia; 17 Health Services, Mercy Health, Heidelberg, VIC Australia; 18 Department of Hepatobiliary and Upper GI Surgery, St. Vincent’s Hospital Melbourne, Melbourne, VIC Australia; 19 Faculty of Health, School of Nursing and Midwifery, Centre for Quality and Patient Safety Research- Epworth Partnership, Deakin University, Geelong, VIC Australia; 20 Sustainability, The Royal Children’s Hospital, Parkville, VIC Australia; 21 Department of Paediatrics, The University of Melbourne, Parkville, VIC Australia; 22 Victorian Virtual Emergency Department, Northern Health, Epping, VIC Australia; 23 Barwon South West Health Service Partnership, Barwon Health, Geelong, VIC Australia; 24 Department of Medicine, Faculty of Medicine, Nursing and Health Sciences, Monash University, Clayton, VIC Australia; 25 Department of Infectious Diseases, Grampians Health Service, Ballarat, VIC Australia; 26 Victorian Infectious Diseases Service, Royal Melbourne Hospital at the Peter Doherty Institute for Infection and Immunity, Parkville, VIC Australia; 27 School of Public Health and Preventive Medicine, Monash University, 553 St Kilda Road, Melbourne, VIC 3004 Australia

**Keywords:** Climate change, Waste, Surveys and questionnaires, Healthcare workers, Sustainability

## Abstract

**Background:**

Healthcare is a significant contributor to greenhouse gas emissions and solid waste. This study evaluated Australian healthcare workers’ knowledge, current behaviours and perceived barriers related to improving healthcare sustainability.

**Methods:**

From November 2023 to January 2024, we conducted an online cross-sectional survey of employees at 12 healthcare organisations in Victoria, Australia. All employees of participating institutions at the time of the survey were considered eligible; students were excluded. The survey link was distributed to all employees via email, and participation was based on self-selection. Our survey included questions from the validated Climate Change Attitudes Survey and Climate and Health Tool, focusing on the domains of awareness, concern and behaviours at home and at work. We also developed original, non-validated questions to explore self-reported barriers to reducing healthcare-associated greenhouse gas emissions and waste, and perceived responsibility for action. Statistical analyses included logistic and linear regression models with cluster-robust standard errors, paired t-tests, Wilcoxon and McNemar tests to compare responses across groups.

**Results:**

We received 2,040 complete and eligible responses. Most respondents were women (1,559, 76%), working in metropolitan locations (1,787, 88%) and employed in the public sector (1,937, 95%). Overall, concern regarding climate change (mean score: 3.29; 5-point Likert scale from 0 to 4) was slightly higher than awareness (mean score: 3.09). Respondents reported taking fewer sustainability actions at work compared to their personal lives (mean score 2.09 versus 2.48, *p* < 0.001). Our original, exploratory questions demonstrated that the three most common barriers to reducing healthcare-associated waste and greenhouse gas emissions were identical: lack of knowledge (39% and 53%, respectively), busyness (26% and 20%) and perceived lack of support from superiors (26% and 19%).

**Conclusions:**

While respondents demonstrated high levels of climate awareness and concern, they faced numerous barriers to pursuing greater sustainability actions at work. The three most frequently reported barriers were a lack of knowledge, a lack of time, and a lack of support. Consistent with previous studies, employees struggled to transfer environmental sustainability practices from their personal lives to the workplace. Our findings offer practical insights into how employee perspectives can shape future strategies to enhance healthcare environmental sustainability.

**Supplementary Information:**

The online version contains supplementary material available at 10.1186/s12913-026-14464-8.

## Introduction

Climate change is the greatest global health threat of the twenty-first century [1], responsible for 13 million deaths annually and projected to increase by 250,000 deaths per year by the 2030s [2]. The healthcare system itself is a significant contributor to climate change. In Australia, healthcare is responsible for 7% of national greenhouse gas (GHG) emissions [3]. Globally, if the healthcare sector were a country, it would be the fifth-largest emitter of GHGs, ranked between Russia and Japan [4]. Healthcare also generates huge volumes of solid waste; in New South Wales, the most populous state in Australia, approximately 8% of all waste stems from healthcare [5]. Importantly, there is a strong waste-GHG relationship in healthcare. For example, many products are single-use and have extensive packaging due to infection control concerns [6], while a significant proportion of healthcare waste is infectious, toxic or radioactive, necessitating specialised (and often more carbon-intensive) disposal or storage [5]. 

Given healthcare’s contribution to climate change, improving environmental sustainability in healthcare could have a profound impact. The World Health Organization (WHO) recently published an operationalised framework for building climate-resilient and low-carbon health systems [7]. This framework aims to provide guidance to the health sector on the systemic transformation necessary to address the challenges posed by climate change. One of the framework’s main pillars is a ‘climate-smart workforce’, which entails ensuring that the healthcare workforce can effectively respond to a dual responsibility: building climate-resilient healthcare systems while also reducing their own carbon footprint. Australia’s inaugural National Health and Climate Strategy also prioritises the need to build health system resilience to the impacts of climate change while supporting reductions in health system GHG emissions [8]. To achieve Australia’s commitment to a net zero health system by 2050 [8, 9], substantial and ongoing transformation within the healthcare sector is required. Importantly, many activities that can reduce healthcare’s carbon footprint, such as environmentally sustainable transport and renewable energy use, also have public health co-benefits and could simultaneously help to reduce future healthcare demand [10]. 

Achieving a successful and rapid transition to environmentally sustainable healthcare will rely on both insights and buy-in from healthcare workers (HCWs) with an intimate understanding of the healthcare system. They can serve as effective partners in both generating and implementing sustainable practices [11]. While prior research has shown HCWs are generally supportive of climate mitigation strategies, they face barriers to implementing sustainable practices relating to insufficient knowledge of climate change, sustainability principles, or the most effective mitigation strategies to pursue [12, 13]. Previous research has often focused on specific professions (e.g. nurses) [13, 14], or specialties [15–17], with limited exploration of transdisciplinary perspectives [18]. The largest Australian survey of HCWs to date, conducted by the Climate and Health Alliance in 2021, highlighted high levels of concern about climate change and its health impacts among almost 900 respondents. However, despite this concern, HCWs felt they lacked sufficient knowledge to discuss these issues with patients, or to advocate for environmentally sustainable workplace practices [19]. While these findings highlight widespread concern, it remains unclear how these feelings vary among different staff subgroups, and a broader analysis of the barriers to improving environmental sustainability in healthcare is needed.

We conducted a large cross-sectional survey of Australian HCWs incorporating diverse experiences from staff across various occupational groups, from both metropolitan and regional areas, and from both public and private healthcare institutions. Our aim was to gather a baseline understanding of HCWs’ knowledge, beliefs, attitudes and current practices regarding climate change and health, and explore differences between HCWs in different occupations and settings. We also sought to investigate perceived barriers to incorporating greater environmental sustainability into healthcare, to inform future policy and research activities.

## Methods

### Study design

This report presents findings from an online survey called the Healthcare Worker Attitudes to Climate Change and Health (WATCH) Study. The WATCH Study employed a mixed-methods approach to explore the beliefs and attitudes of Australian HCWs regarding sustainable practices in emissions and waste reduction within the healthcare sector. We report findings in adherence with the guidelines outlined in the STROBE Checklist for cross-sectional studies (STROBE Statement, Appendix File 2, pp16-18) [20]. 

### Participants

HCWs were recruited from twelve healthcare organisations across Victoria, Australia, encompassing both private and public providers, and regional and metropolitan sites. For the purposes of the WATCH Study, ‘healthcare workers’ included all employees regardless of their profession. We included non-clinical staff (e.g. from engineering, research and administration) in recognition that environmental sustainability in healthcare involves *all* HCWs, not just those in clinical roles like nursing, allied health or medicine. The study utilised a self-selection sampling method, with participants voluntarily responding to an open invitation to complete the survey. From November 2023, investigators at participating sites received a standardised invitational email, an e-newsletter template and a promotional flyer that could be customised for their organisation. All employees at participating organisations were invited to complete a survey either via all-staff CEO emails or organisation-wide e-newsletters. Additionally, posters were placed in non-patient areas (e.g. staff tea-rooms) or included as advertisements on staff computer screensavers. Sites were prompted to increase communication efforts after two and four weeks. All promotional materials included either a link or a QR code directing individuals to the online survey platform. All participants were given the opportunity to participate in a randomised draw to receive one of five digital gift cards.

Eligibility criteria included all adults employed at a participating study site at the time of recruitment; healthcare students were excluded. The survey was conducted in English. We considered participants who initiated but did not complete the survey to have withdrawn their consent and did not use their data. The survey was accessible from 8 November 2023 to 8 January 2024.

### Procedures

Survey data were collected and managed through REDCap, a secure web-based platform [21]. The online survey (Main Survey, Appendix File 1 pp3-15) included questions from two previously validated instruments. The Climate and Health Tool consisted of five psychometrically evaluated scales measuring awareness, motivation, concern, behaviours at work and behaviours at home [14, 22]. Respondents were asked to rate items on a five-point Likert scale (scored from zero to four), with higher scores indicating higher levels of the construct. Prioritising brevity of our survey, we decided not to include the motivation scale. We also slightly modified some questions to better suit an Australian audience (e.g. replacing ‘Lyme disease’ with ‘Japanese encephalitis’ as an example of a condition influenced by climate change).

We included five structured questions from the Climate Change Attitudes Survey (CCAS) to measure climate beliefs [23]. Our survey also included further original semi-structured questions to explore how HCWs viewed climate change action compared to other healthcare priorities, as well as their perceived responsibilities for, and barriers to, reducing healthcare-associated waste and GHG emissions. These questions were designed based on existing literature and piloted with clinical and non-clinical staff representatives plus environmental sustainability officers not involved in their design, but were not psychometrically evaluated due to time and resource constraints. To capture data on HCWs thoughts, we also asked participants for their ideas about reducing healthcare-associated waste and greenhouse gas emissions with two free-text response questions: ‘What ideas do you have for reducing healthcare-associated waste in your role?’ and ‘What ideas do you have for reducing healthcare-associated greenhouse gas emissions in your role’.

Finally, we included demographic questions on age, gender, healthcare organisation and work experience. The survey was piloted with more than a dozen clinical and non-clinical healthcare staff to inform survey length, layout and readability.

### Statistical analysis

All statistical analyses were performed using Stata software (ver. 18, StataCorp, LLC, College station, Texas, USA) at alpha = 0.05 significance level. Respondent characteristics and outcomes were summarised using Mean (SD) / Median (IQR) for numerical variables, and using frequency (percent) for categorical variables. For main outcome measures of the study, the 95% confidence intervals (CIs) were computed using binomial exact procedure. Missingness was examined as a binary outcome (1 = missing, 0 = non-missing) in a multivariable logistic regression model, with background factors as covariates and organisational clustering accounted for using cluster-robust standard errors. No significant differences were observed between missing and non-missing data, so subsequent analyses were based on complete case data. The survey response rate was calculated using aggregate employee data obtained from participating organisations’ human resource departments or most recent Annual Report. We made a conservative assumption that every employee at each organisation received and viewed an invitation to complete the survey.

We compared CCAS score, climate awareness, concern, and behaviours (at work and at home) using a logistic regression model. To account for clustering within study sites, the model included an effect for organisational affiliation and utilised cluster robust standard error estimation. Accordingly, Odds Ratios (ORs) and their 95% CIs are presented. For paired analyses comparing outcomes scores, paired t-tests and Wilcoxon signed-rank test were used. For paired comparisons across binary outcomes, McNemar tests were performed and results are reported as paired ORs (95% CI). In addition, to compare barriers score across background variables’ groups, due to non-normality of the scores, a linear regression model was used after log transformation of the scores. The organisation clustering effect was considered utilising cluster robust standard error, and geometric means with 95% CIs were computed for the scores to describe the distribution. Free-text data were analysed thematically, primarily using an inductive approach following the methodology outlined by Braun & Clarke [24]. 

## Results

Based on aggregated employee data, we estimated the 12 healthcare organisations had a total of 97,125 employees. We received 2,301 survey responses, of which 2,111 were complete and 2,040 met eligibility criteria and were included in analyses. The estimated response rate was 2·1% (2,040 / 97,125) overall, with individual site response rates ranging from 0·2% to 11% (median 1·8%, IQR 1·1–3·4%). The mean time to survey completion was 12 min. Most respondents were women (*n* = 1,559, 76·4%), in line with the female predominance of the surveyed workforce (71% among the nine organisations where gender data for all staff were available). Nurses and midwives were the most frequent profession (*n* = 611, 30·0%), followed by allied health workers (*n* = 436, 21·4%) and doctors (*n* = 331, 16·2%) (Table 1). Over half of respondents had more than 10 years’ work experience in their current profession (*n* = 1,141, 55·9%). The majority of responses came from staff working in the public healthcare sector (*n* = 1,937, 95·0%) and at metropolitan sites (*n* = 1,787, 87·7%).

**Table 1:** Demographics of all participants, (*n* = 2,040)

Demographics	Frequency
**Total**	2,040 (100%)
**Gender**	
Women Men Non-binary / Gender diverse Not listed Prefer not to say	1,559 (76·4%) 388 (19·0%) 11 (0·5%) 4 (0·2%) 78 (3·8%)
**Age (in years)**	
18–29 30–39 40–49 50–59 60+	245 (12·0%) 559 (27·4%) 486 (23·8%) 496 (24·3%) 254 (12·5%)
**Profession**	
Allied Health Clerical/Administrative Doctor Nurse/Midwife Other^*^	436 (21·4%) 323 (15·8%) 331 (16·2%) 611 (30·0%) 339 (16·6%)
**Work Experience**	
≤ 10 years > 10 years	899 (44·1%) 1141 (55.9%)
**Sector^**	
Public Private	1,937 (95·0%) 102 (5·0%)
**Geographical location^**	
Metropolitan Regional	1,787 (87 ·7%) 252 (12·3%)

Data are n (%)

*Other professions included but were not limited to pharmacists (*n* = 79), clinical scientist/technicians (*n* = 67), researchers (*n* = 36), engineers (*n* = 20), and patient services assistants (*n* = 18)

^Data not available for one respondent

Most respondents (*n* = 1,635, 80·1%) considered it moderately or extremely important that their healthcare organisation prioritised reducing its contribution to climate change (Appendix Table 1). When compared against five other competing healthcare priorities, such as addressing workforce shortages or reducing wait-times for patients, 54·5% of respondents rated addressing climate change as above average importance (Appendix Table 1). This was accompanied by moderate to high levels of concern about the impacts of climate change (mean score of 3·29; 3= “Moderately”, 4= “Extremely”), with women (3.37) reporting higher levels of concern than men (3.07; *p* < 0.001) (Appendix Table 3) and metropolitan workers (3.30) reporting higher levels of concern than their regional counterparts (3.17; *p* = 0.015) (Appendix Table 3). Respondents reported moderate to high levels of climate awareness overall (mean score 3·09; 3= “Moderately”, 4= “Extremely”). Awareness was highest for recognising that climate change is occurring (two questions, mean 3·40), but significantly lower for understanding climate change’s impacts on health (two questions, mean 3·15) or recognising the healthcare system’s contribution to climate change (one question, mean 2·32; *p* < 0·001 for differences between each set of questions) (Appendix Table 2).

**Table 2:** Comparison of overall frequency of barriers for addressing waste and greenhouse gas emissions, according to geographic location and level of work experience

Barriers	Regional vs. Metropolitan				≤ 10yrs Work experience vs. >10yrs Work experience			
	Waste OR (95%CI)	*p*-value	GHG emissions OR (95%CI)	*p*-value	Waste OR (95%CI)	*p*-value	GHG emissions OR (95%CI)	*p*-value
Don’t know what to do	0·98 (0·71 − 1·35)	0·886	0·84 (0·56 − 1·24)	0·372	1·27 (1·07 − 1·50)	**0**·**005**	1·25 (1·06 − 1·48)	**0**·**010**
Too busy	1·01 (0·77 − 1·32)	0·932	1·23 (0·83 − 1·82)	0·293	1·02 (0·89 − 1·18)	0·734	1·01 (0·89 − 1·14)	0·928
Don’t believe changes would be supported by superiors	1·01 (0·70 − 1·44)	0·976	1·04 (0·65 − 1·64)	0·883	0·93 (0·76 − 1·13)	0·469	0·85 (0·64 − 1·13)	0·269
More pressing concerns	1·57 (1·10 − 2·22)	**0**·**012**	1·68 (1·12 − 2·51)	**0**·**012**	1·06 (0·89 − 1·26)	0·550	1·19 (0·97 − 1·45)	0·092
Not confident to act	1·16 (0·91 − 1·48)	0·239	1·13 (0·85 − 1·50)	0·408	1·34 (1·02 − 1·78)	**0**·**038**	1·16 (0·88 − 1·54)	0·289
Inconvenient	0·77 (0·67 − 0·89)	**0**·**001**	0·96 (0·76 − 1·23)	0·760	1·25 (1·08 − 1·45)	**0**·**003**	1·17 (0·87 − 1·57)	0·299
COVID-19 fatigue	1·36 (0·87 − 2·14)	0·177	1·73 (1·15 − 2·61)	**0**·**009**	1·15 (0·83 − 1·60)	0·405	1·16 (0·89 − 1·50)	0·282
I don’t believe I could make a difference	0·90 (0·69 − 1·17)	0·426	1·16 (0·92 − 1·45)	0·210	1·35 (1·01–1·79)	**0**·**041**	1·62 (1·30 − 2·01)	**< 0**·**001**
Choose to spend time on other important issues	1·24 (0·65 − 2·37)	0·518	1·52 (0·94 − 2·44)	0·086	1·20 (0·95 − 1·51)	0·132	1·21 (0·77 − 1·88)	0·410
Other	0·89 (0·51 − 1·54)	0·673	0·80 (0·44 − 1·45)	0·458	0·80 (0·65 − 0·98)	**0**·**032**	0·68 (0·55 − 0·83)	**< 0**·**001**

**Table 3:** Obstacles and underlying issues relating to improving healthcare sustainability

Obstacle	Underlying issue(s)
“I don’t know what to do” the most cited barrier, especially among less experienced staff	Lack of knowledge regarding how to address healthcare sustainability
Staff frequently reported feeling too busy or having more pressing concerns	Prioritising sustainability in healthcare was challenging, even for this motivated cohort of respondents
Perception that sustainability changes would not be supported by superiors	In addition to a lack of knowledge, ability to affect change is impaired by lack of perceived senior support
HCWs taking more sustainability actions at home compared to at work	Lack of agency or authority for staff to translate behaviours into a work context, may be related to perceived lack of support
Additional challenges for smaller/regional hospitals	Smaller pool of staff available to take on additional responsibilities
Greater familiarity with and sense of responsibility for waste compared to GHG emissions	GHG emissions are more nebulous than waste and less easily understood or identified. HCWs feel they have less control over GHG emissions
Strong awareness of climate change, with lower awareness of its health impacts or of the healthcare sector’s contributing role	Concern and awareness around climate change is not translating into equivalent awareness of its health impacts, or the large potential positive impacts of improved healthcare sustainability

In terms of behaviours, respondents were less likely to report taking actions at work to address climate change and waste (mean score of 2·09; 2= “Rarely”, 3= “Sometimes”) compared to their personal lives (2·48; *p* < 0·001). More experienced HCWs (more than 10 years’ work experience) reported a higher frequency of sustainability behaviours in both home (2.55) and work settings (2.13) compared to less experienced workers (2.39; *p* < 0.001, and 2.04; *p* = 0.006, respectively). Similarly, sustainability behaviours at work and at home were more common with each incremental increase in age (*p* < 0.001 and *p* = 0.005, respectively) (Appendix Table 3). Doctors and nurses exhibited the highest frequency of sustainability behaviours in both settings (Appendix Table 3).

Responses to the CCAS questions indicated high levels of belief in climate change and the importance of action, with an overall mean score of 4·36 out of 5 (Appendix Table 4). Women had higher CCAS scores than men (4.41 versus 4·24; *p* < 0·001) and doctors had the highest mean score (4·44; *p* < 0·001), however there were no meaningful differences seen between other subgroups (Appendix Table 4).

**Fig. 1:**
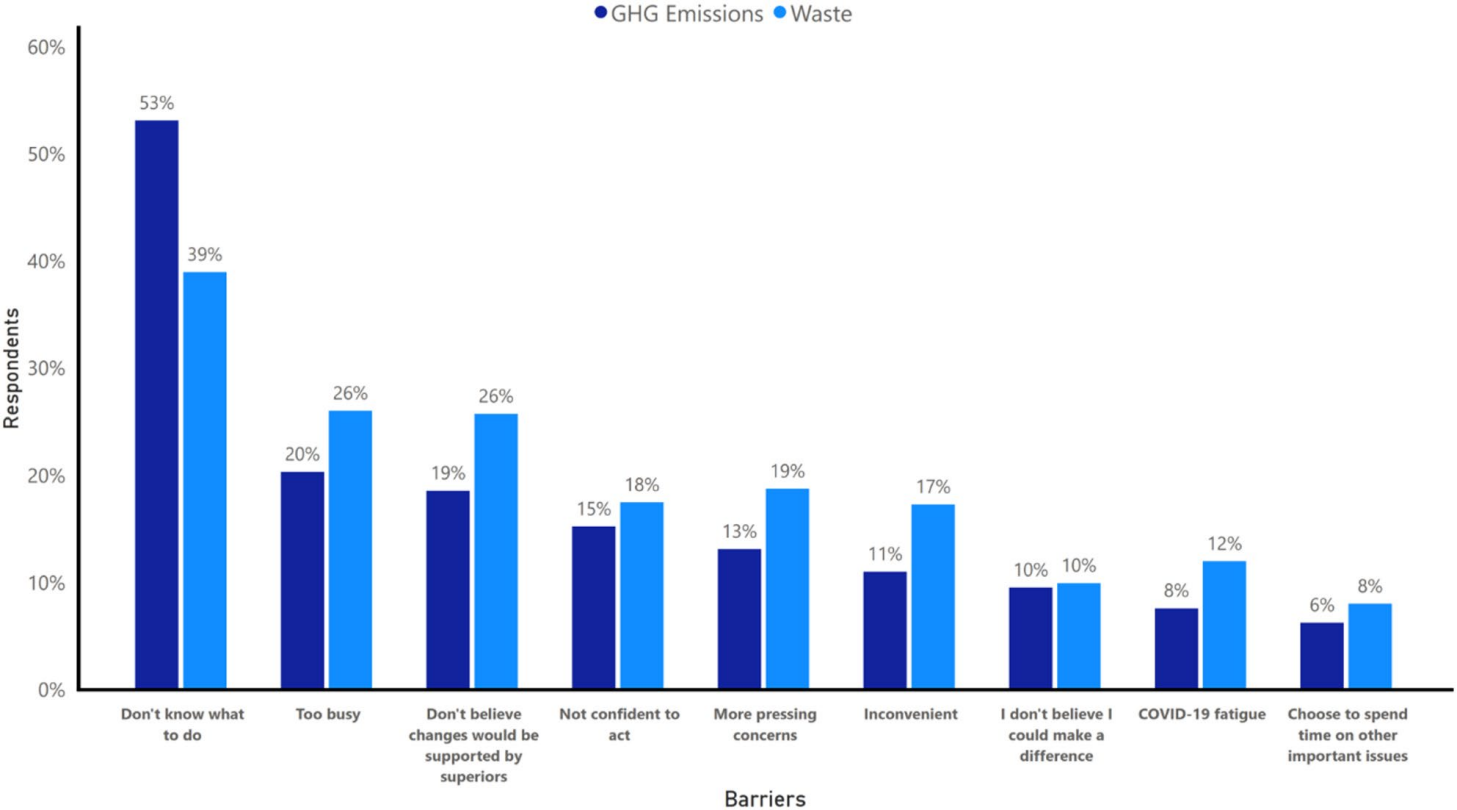
Barriers to addressing healthcare-associated waste and greenhouse gas emissions. 20% and 10% of respondents also selected ‘Other’ when reporting on their perceived barriers for waste and GHG emissions, respectively

Most HCWs expressed a desire to do more in their work role to reduce waste (*n* = 1,616, 79·2%) and GHG emissions (*n* = 1,383, 67·8%) (Appendix Fig. 1). However, they faced several barriers. The most common perceived barriers were: ‘I don’t know what to do’, ‘I am too busy’, and ‘I don’t believe changes would be supported by my superiors’ (Fig. 1). A lack of knowledge was a more common barrier to addressing GHG emissions (53·1%) compared to waste (39·0%; p < 0·001) (Appendix Table 5). Doctors identified the highest number of barriers on average (2·02), followed by allied health workers (1·70), nurses (1·69), and clerical/administration staff (1·37; p < 0·001) (Appendix Table 6). Doctors were the most likely to report ‘more pressing concerns’, ‘inconvenient’, ‘choose to spend time on other important issues’, and ‘too busy’ as barriers to reducing both waste and GHG emissions (all p < 0·05) (Appendix Table 7). In contrast, nurses and midwives were most likely to report fatigue related to the COVID-19 pandemic as a barrier to reducing waste (14·1%; p < 0·001) and GHG emissions (10·3%; p < 0·001) (Appendix Table 7).

The frequency of certain barriers also varied by geographic location and work experience. HCWs in regional settings were more likely to report ‘more pressing concerns’ and ‘COVID-19 fatigue’ as barriers to reducing waste (ORs 1·57 and 1·36, respectively) and GHG emissions (ORs 1·68 and 1·73, respectively), although the association between ‘COVID-19 fatigue’ and waste was not statistically significant (Table 2). Less experienced (≤ 10 years) HCWs were more likely to report ‘I don’t know what to do’ and ‘I don’t believe I could make a difference’ as barriers to both waste (ORs 1·27 and 1·35, respectively) and GHG emission reductions (ORs 1·25 and 1·62, respectively). In contrast, the belief that changes would not be supported by superiors was shared more evenly no matter the level of HCW experience (OR 0·929 [p = 0·469] for waste and OR 0·852 [p = 0·269] for GHG emissions) (Table 2).

Hospital Executives were the group most frequently identified by HCWs as responsible for taking action to reduce both waste (83·6%) and GHG emissions (84·7%). HCWs were more likely to view themselves as responsible for addressing waste compared to reducing GHG emissions (81·0% versus 60·5%; *p* < 0·001). Whereas respondents placed the responsibility for reducing waste relatively equally across all response options, for GHG emissions perceived responsibility increased as one moved further away from the individual level (Appendix Fig. 2).

A total of 1157 and 592 free-text responses were analysed in response to questions prompting HCWs suggestions for strategies to address healthcare-associated waste and GHGs respectively, with three major themes discovered in responses to each question. Waste: Improved processes and systems for waste disposal, Encouraging a sustainable culture of reduce/reuse/recycle, Organisational environmental leadership (Appendix Table 8). GHGs: Normalising sustainable practices in the culture of the workplace, Energy efficient solutions to the workplace, and Bridging the gap between individual and systemic changes (Appendix Table 9).

## Discussion

We conducted the most comprehensive survey to date about Australian HCWs’ knowledge and attitudes towards climate change, gathering over 2,000 responses from a diverse range of professions and geographic locations. Consistent with previous research in both global and Australian contexts, our respondents are highly concerned about climate change and want to be doing more at work to promote environmentally sustainable healthcare, yet identified numerous barriers to achieving this goal [11, 12, 19]. This existing enthusiasm for healthcare sustainability could be harnessed to promote the development of a climate-resilient and low-emissions health sector, in line with Australia’s National Health and Climate strategy [8]. However, our findings suggest that the ability of HCWs to address environmental sustainability is currently hindered by a number of barriers, primarily relating to either a lack of knowledge, a lack of time, or a lack of institutional support.

The primary barrier reported by HCWs in our study was insufficient knowledge, with ‘I don’t know what to do’ the most commonly identified obstacle for addressing both healthcare-associated waste and GHG emissions. Similarly, several respondents suggested they were not sure of the best approach for reducing waste and GHG emissions in free-text responses. While a lack of knowledge has been widely reported in many previous HCW studies as either the commonest barrier to greater sustainability action [17, 19, 25], or within the top few barriers [11, 12, 16, 18, 25–27], its prominence in our study is notable. Respondents cited a lack of knowledge almost twice as often as the next most common barriers: being too busy or having competing priorities. Additional education, training and awareness around sustainable workplace practices was also a common thread in free-text responses. In terms of providing greater HCW education, we note that the Australian Commission for Safety and Quality in Healthcare has recently developed a pilot module on Environmental Sustainability and Climate Resilience in Healthcare [28], a positive step towards broader health and climate training for all Australian HCWs. In the future this module may be implemented and assessed alongside existing national quality standards. Additionally, a focus on the health impacts of climate change has recently been added to the Australian Medical Council standards for medical schools and prevocational medical training [29], and there are calls from peak bodies for similar content to be added to Australian nursing degrees [30]. The WHO’s 2023 operational framework for climate resilient and low-carbon health systems also emphasises the important role of HCW education, nominating the percentage of HCWs undergoing training on both sustainability and climate resilience as key measurable indicators for countries to track [7]. Additional training in sustainable healthcare is currently available, for instance through providers such as the Centre for Sustainable Healthcare (https://sustainablehealthcare.org.uk/), however HCWs face both financial and time constraints in accessing these formal training opportunities. Improving access to training and education on sustainable healthcare, especially for HCWs already expressing a strong desire to be doing more, can address knowledge gaps and help to develop and empower future leaders in this field.

Behind insufficient knowledge, the next most frequently identified barriers were being too busy and not believing changes would be supported by superiors. A sense of excessive busyness among HCWs is unsurprising given this barrier has previously been reported in the literature [11, 31], as well as the fact that our survey was conducted in 2023 as the healthcare system emerged from the COVID-19 pandemic that had caused significant upheaval of the national health workforce [32] and increased staff workloads and work-life imbalance [33]. One solution to overcome a lack of time is to provide motivated HCWs with dedicated, protected time during business hours to pursue sustainability activities. The ‘Net Zero Leads’ program in New South Wales, Australia, provided funding to back-fill the clinical roles of identified sustainability champions one day per week, allowing them to research, develop and embed low-carbon models of care within their health services [34]. In a constrained financial environment, it can be challenging for healthcare organisations or governments to source funds to support such initiatives, however environmental sustainability activities frequently lead to cost-savings through increasing efficiencies or reducing unnecessary resource consumption [35]. Two other key barriers identified by WATCH were a lack of perceived support as well as a lack of agency within the work environment. A similar disconnect between individual capacity and organisational leadership on environmental sustainability was present in free-text responses. These finding reinforce the important role of healthcare leadership – the first component listed in the WHO Framework [7] – in championing environmental sustainability and demonstrating a commitment to taking action. Interestingly, more experienced HCWs were less likely to report a lack of either knowledge or confidence as barriers, but – although the difference was not statistically significant – they had lower perceptions of the senior support they would receive. This cohort may represent a potential pool of engaged HCWs with a strong desire to implement positive changes, but are held back by a real or perceived lack of support from their organisational leadership.

Table 3 provides a summary of key barriers preventing HCWs from taking greater action on waste and GHG emissions; it will be crucial for healthcare organisations and policymakers to address these obstacles as they strive to embed greater environmental sustainability within healthcare.

We identified notable differences in HCW responses on specific topics, providing potential insights into how to optimise future HCW engagement with healthcare sustainability efforts. First, while HCWs demonstrated high awareness of climate change occurring, their awareness decreased significantly when questions focused on the associations between climate change and health, and even more so when considering healthcare’s contribution to climate change. This suggests that while HCWs are presently aware of climate change in general, they may not fully recognise its relevance to their work. Second, when assessing individual behaviours to address climate change, respondents were less likely to take actions at work compared to in their personal lives, potentially implying they feel they have less control or influence over environmental actions in their work environment. Third, in comparing responses regarding waste and GHG emissions, HCWs consistently reported greater knowledge and sense of personal responsibility for tackling waste, whereas they felt less confident in their ability to address GHG emissions, which they tended to view as the responsibility of others. These differences may reflect both the more visible nature of waste, as well as an incomplete understanding of the sources of GHG emissions, as waste itself contributes to emissions through the resources required to manufacture, transport and dispose of (frequently single-use) items. With recent evidence estimating that Scope 3 (indirect) emissions – including purchased goods and services (such as medical supplies, equipment and pharmaceuticals), capital goods, and patient and visitor travel – account for up to 75% of total healthcare GHG emissions [36], our findings again underscore the importance of enhancing HCWs’ knowledge and awareness of the various contributions to healthcare’s carbon footprint. Finally, having ‘more pressing concerns’ and ‘COVID-19 fatigue’ were barriers more commonly reported by respondents from regional sites, potentially reflecting the smaller pool of HCWs in these organisations and staff having to shoulder more responsibilities.

Direct comparisons to existing literature are challenging due to the lack of use of psychometrically evaluated measurement tools, variability in study design, and focus on distinct occupational groups or research topics. For instance, the previous largest survey of HCWs in Australia used the ‘Six Americas’ framework to classify attitudes towards climate change; it also focused on whether HCWs were seeing climate impacts in their communities and whether they felt confident discussing climate change with their patients [19, 37]. In terms of available comparisons, our respondents’ mean scores for domains of the Climate and Health Tool were similar to those reported among a subgroup of licensed nurses from the United States in 2021 [14], although nurses in our study reported higher awareness and concern. The main barriers we identified align with those reported in a recent United Kingdom study of National Health Service and social care staff, which found strong motivation for environmentally sustainable changes but a lack of agency due to competing priorities, poorly perceived leadership and a lack of understanding around implementation [38]. Similar findings of strong motivation coupled with limited practical understanding, and perceptions that proposed changes lack institutional support, have also been observed in other healthcare settings [17, 25, 26, 39]. Our observation that sustainability behaviours in HCWs’ home environments are not being transferred into professional settings is consistent with prior research [14, 15, 17, 40]. Evidence from the behaviour change literature points to the importance of capability, opportunity and motivation for implementation of behaviour change [41]. Our findings suggest that we could leverage from HCWs’ existing motivation (as evidenced by their home behaviours) to increase uptake of sustainable workplace practices. Healthcare organisations should therefore strive to increase their HCWs’ capacity (e.g. through education and training) and opportunities (e.g. through formal support for sustainability initiatives) to translate their staff’s motivation levels into meaningful improvements in healthcare environmental sustainability [13, 38]. 

A strength of this research is the high number of respondents from twelve different healthcare organisations, representing a wide range of professions and settings, both metropolitan and regional. A central limitation of our study is the low overall response rate, calculated at 2·1%, although this is likely an underestimate as we assumed every single employee viewed an invitation to our survey. While our response rate is comparable to other studies that also used broad promotional strategies [31], it is lower than the response rates of 10–40% achieved via more targeted recruitment strategies (e.g. limited to specific professional groups or existing mailing lists) [17, 25–27]. Consequently, our respondents may not completely reflect the broader workforce – for instance 76% of our respondents were women, compared to 71% in participating organisations – and are likely skewed towards HCWs who are more enthusiastic and engaged with this topic. It is therefore particularly noteworthy that even among a presumably motivated cohort, there were knowledge gaps regarding both climate change’s impact on health and healthcare’s carbon footprint, as well as a low perceived sense of agency to enact change within the healthcare system. Another limitation is that we were unable to control for participants responding to the survey more than once. While the use of an anonymous public survey link was seen as a method for boosting engagement and minimising reporting bias, it is plausible that some participants may have provided multiple responses. Further, due to sparse observations for many occupations, we were unable to implement occupation-level clustering or multilevel modelling, potentially leaving some intra‐organisational heterogeneity unmodeled in our analysis. Additionally, as highlighted above, survey questions around barriers to action on waste and GHG emissions –were original and have not been formally validated. Nevertheless, we feel these provide helpful exploratory findings, that were frequently supported by the existing published literature. A further limitation was that due to uncertainty regarding the real-world practical significance of differences in scores in the validated survey instruments we used, we instead relied on reporting statistical significance. Finally, the majority of staff at our twelve participating healthcare organisations are involved in hospital-based care, so we are unable to extrapolate our results to community healthcare settings. Future qualitative research could complement our anonymous survey findings, contributing to a more holistic view of staff perspectives on environmental sustainability in healthcare.

## Conclusions

This study highlights key findings about Australian HCWs’ attitudes and behaviours toward climate change and sustainability. While respondents expressed strong concern and motivation to act, their ability to engage in environmentally sustainable practices was limited by insufficient knowledge, time constraints, and a perceived lack of organisational support. Notably, HCWs felt more responsible for addressing waste than greenhouse gas emissions and were more likely to take sustainability actions in their personal lives than at work. These findings suggest that despite high levels of awareness, there remains a disconnect between individual motivation and workplace implementation. Looking forward, healthcare organisations and policymakers must prioritise education, leadership engagement, and structural support to empower HCWs and embed sustainability into routine practice. Strengthening workforce capability and opportunity will be essential to achieving a climate-resilient, low-emissions healthcare system aligned with national and global health and climate goals.

## Supplementary Information

Below is the link to the electronic supplementary material.

Supplementary Material 1

## Data Availability

Deidentified participant data will be made available upon reasonable request to the corresponding author.

## Abbreviations

CCAS: Climate Change Attitudes Survey
CI: Confidence interval
GHG: Greenhouse gas
HCW: Healthcare worker
IQR: Interquartile range
OR: Odds ratio
SD: Standard deviation
WATCH Study: Healthcare Worker Attitudes to Climate Change and Health Study
WHO: World Health Organization
