# Supplementary material for: Australian healthcare workers’ perspectives, practices, and perceived barriers to a more environmentally sustainable health system: a cross-sectional survey
Source: BMC Health Serv Res. 2026 Mar 31;26:677. doi: 10.1186/s12913-026-14464-8 (PMC13162466; doi:10.1186/s12913-026-14464-8)
Supplement: Supplementary file 1 — Supplementary Material 1 [file 12913_2026_14464_MOESM1_ESM.docx]

**Supplementary Appendix: *Australian healthcare workers’ perspectives, practices, and perceived barriers to a more environmentally sustainable health system: a cross-sectional survey* – BMC Health Services Research**

**Table of Contents**

[**Appendix File 1: Main Survey** 4](#_Toc213324249)

[**Appendix File 2: STROBE Statement** 17](#_Toc213324250)

[**Appendix Table 1: Proportion of respondents who prioritised institutional action on climate change above other workplace issues, by gender, age group, occupation, work experience, sector, and location** 20](#_Toc213324251)

[**Appendix Table 2: Paired comparison of scores for awareness about climate change, awareness of the health impacts of climate, and awareness of healthcare contribution to climate change, for the entire cohort** 21](#_Toc213324252)

[**Appendix Table 3: Average scores for different domains of the Climate and Health Tool*** 22](#_Toc213324253)

[**Appendix Table 4: Mean score for Climate Change Attitudes Survey (CCAS) questions by gender, age group, profession, work experience, sector, and geographical location** 23](#_Toc213324254)

[**Appendix Table 5: Pairwise comparison of responses selecting not knowing what to do about waste versus greenhouse gas (GHG) emissions, for the entire cohort** 24](#_Toc213324255)

[**Appendix Table 6: Variance in average frequency of reported barriers for waste or greenhouse gas (GHG) emissions by gender, profession, work experience and geographical location** 25](#_Toc213324256)

[**Appendix Table 7: Comparison of reported barriers for waste and greenhouse gas (GHG) emissions, by profession** 26](#_Toc213324257)

[**Appendix Table 8: Thematic analysis of 1157 responses to “What ideas do you have for reducing healthcare-associated waste in your role?”, including illustrative quotes** 27](#_Toc213324258)

[**Appendix Table 9: Thematic analysis of 592 responses to the question “What ideas do you have for reducing healthcare-associated greenhouse gas emissions in your role?”, including illustrative quotes** 29](#_Toc213324259)

[**Appendix Figure 1: Perceived responsibility for addressing healthcare-associated waste and greenhouse gas emissions** 31](#_Toc213324260)

[**Appendix Figure 2: Respondents expressing a desire to do more to address waste and greenhouse gas emissions in their role** 32](#_Toc213324261)

# **Appendix File 1: Main Survey**


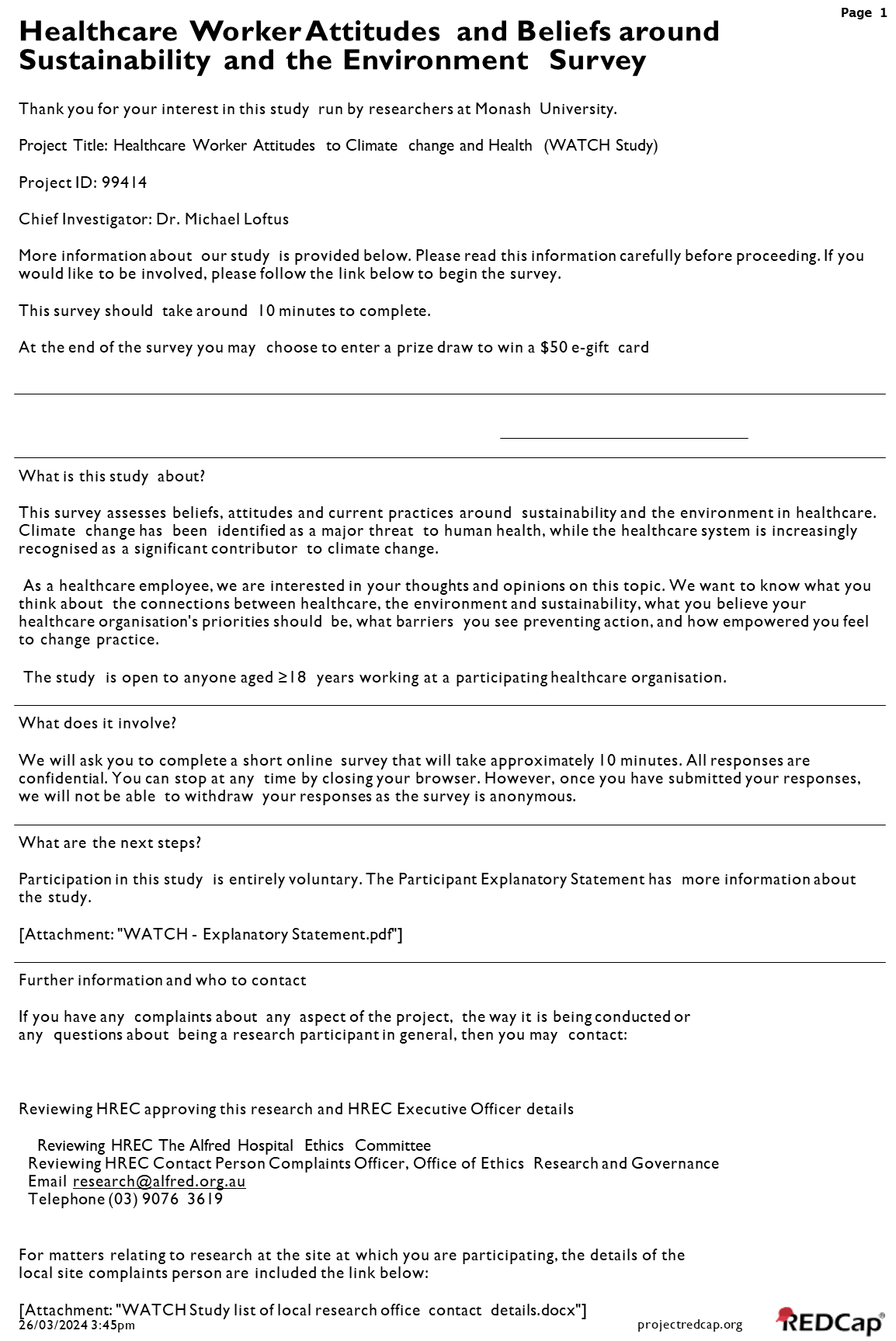


More information about our study is provided below. Please read this information carefully before proceeding. If you

would like to be involved, please follow the link below to begin the survey.

**Healthcare Worker Attitudes and Beliefs aroundSustainability and the Environment Survey**

Thank you for your interest in this study run by researchers at Monash University.

**Page 1**

What does it involve?

We will ask you to complete a short online survey that will take approximately 10 minutes. All responses are

confidential. You can stop at any time by closing your browser. However, once you have submitted your responses,

we will not be able to withdraw your responses as the survey is anonymous.

What are the next steps?

Participation in this study is entirely voluntary. The Participant Explanatory Statement has more information about

the study.

[Attachment: "WATCH - Explanatory Statement.pdf"]

Further information and who to contact

If you have any complaints about any aspect of the project, the way it is being conducted or

any questions about being a research participant in general, then you may contact:

Reviewing HREC approving this research and HREC Executive Officer details

Reviewing HREC The Alfred Hospital Ethics Committee

Reviewing HREC Contact Person Complaints Officer, Office of Ethics Research and Governance

Email [research@alfred.org.au](mailto:research@alfred.org.au)

Telephone (03) 9076 3619

For matters relating to research at the site at which you are participating, the details of the

local site complaints person are included the link below:

[Attachment: "WATCH Study list of local research office contact details.docx"]

26/03/2024 3:45pm

projectredcap.org

**
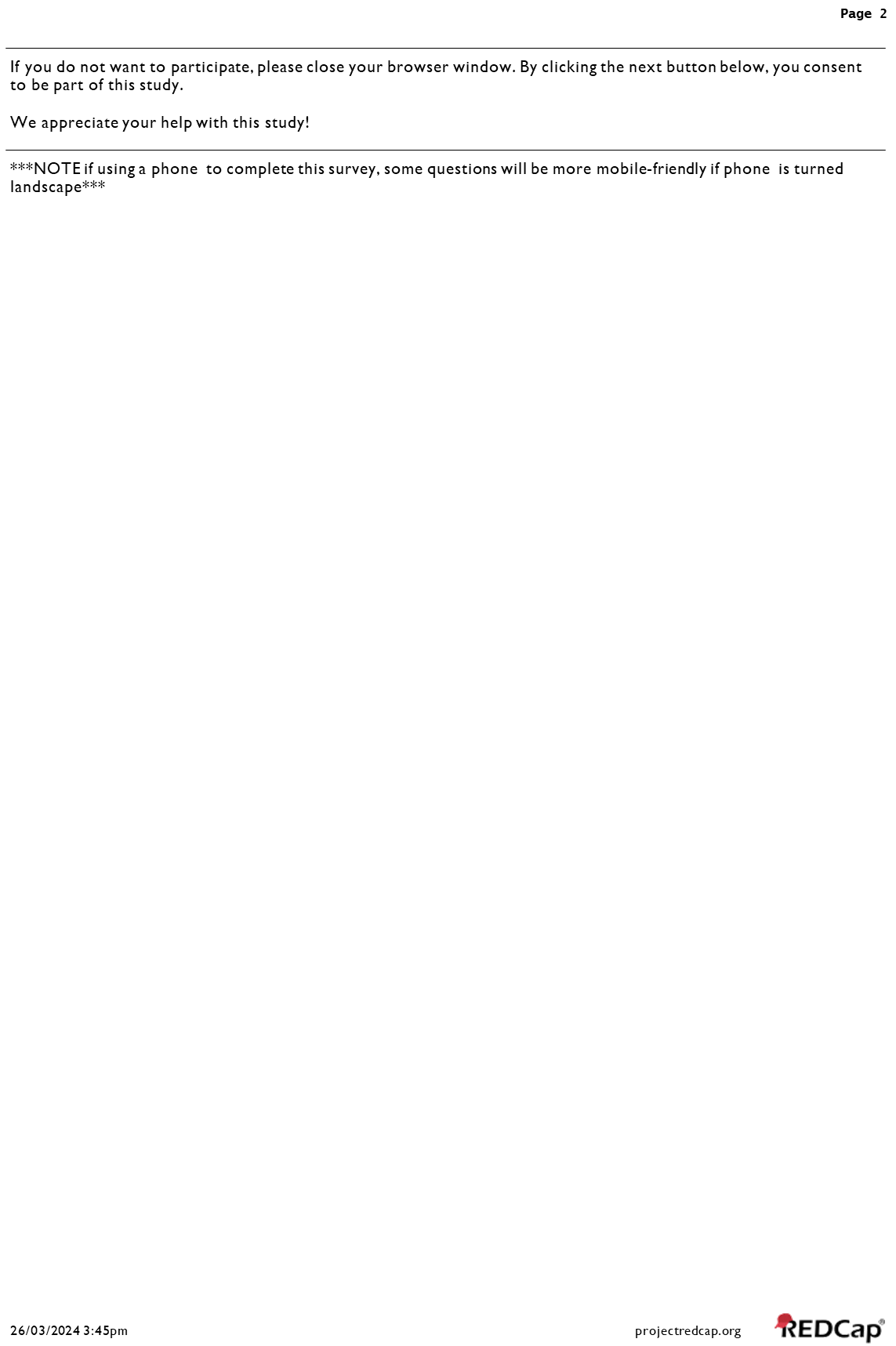
**

**
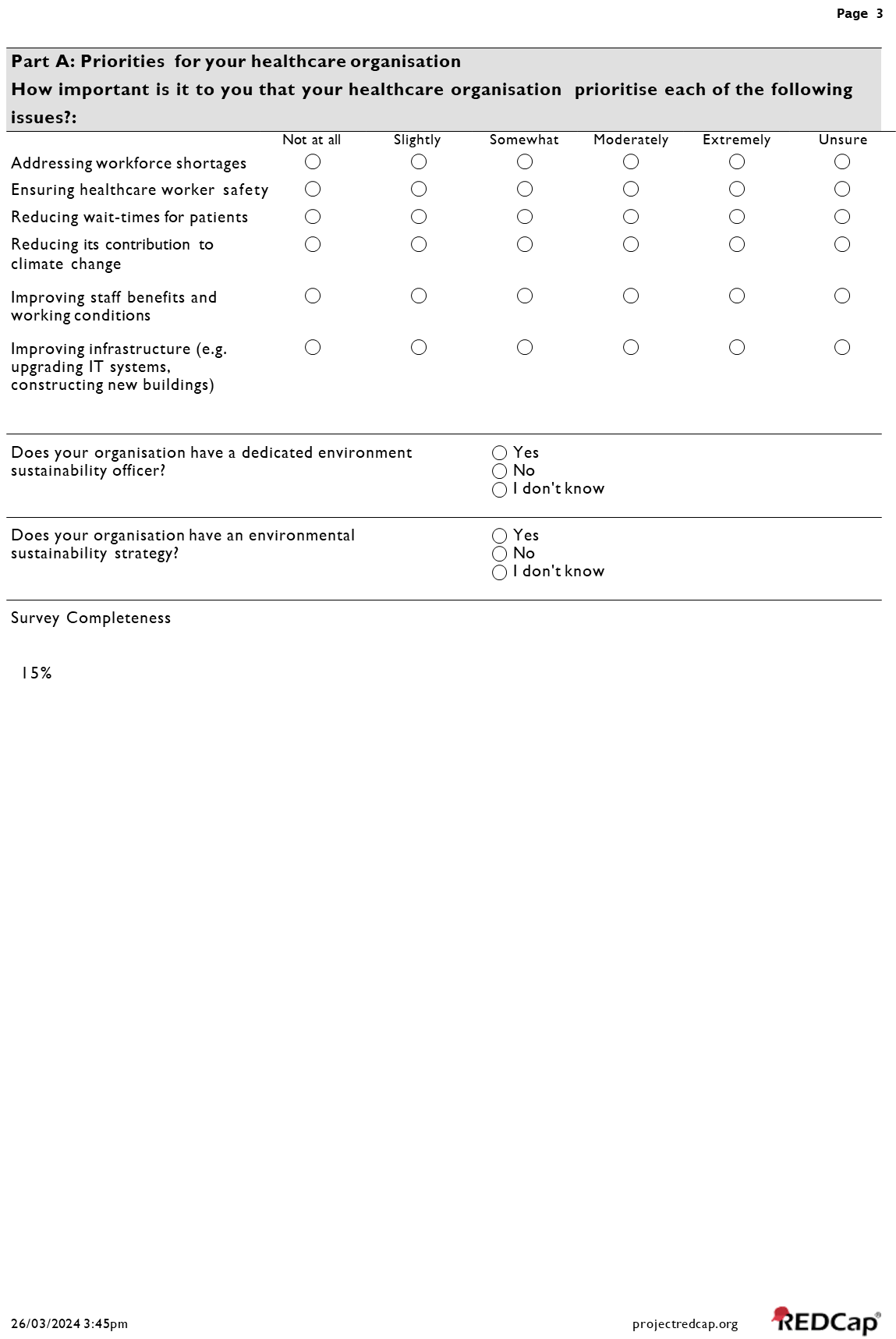
**

**
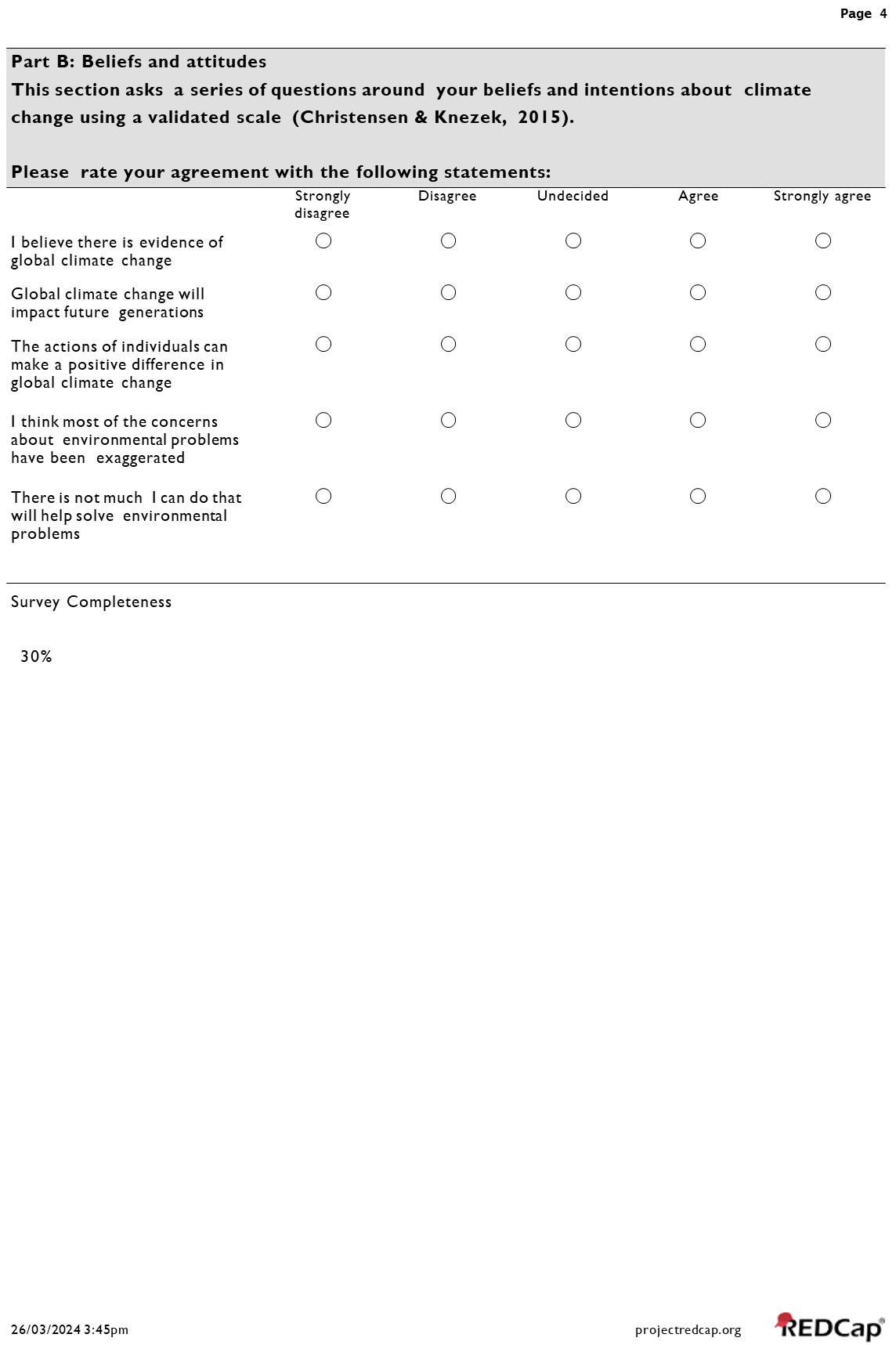
**

**
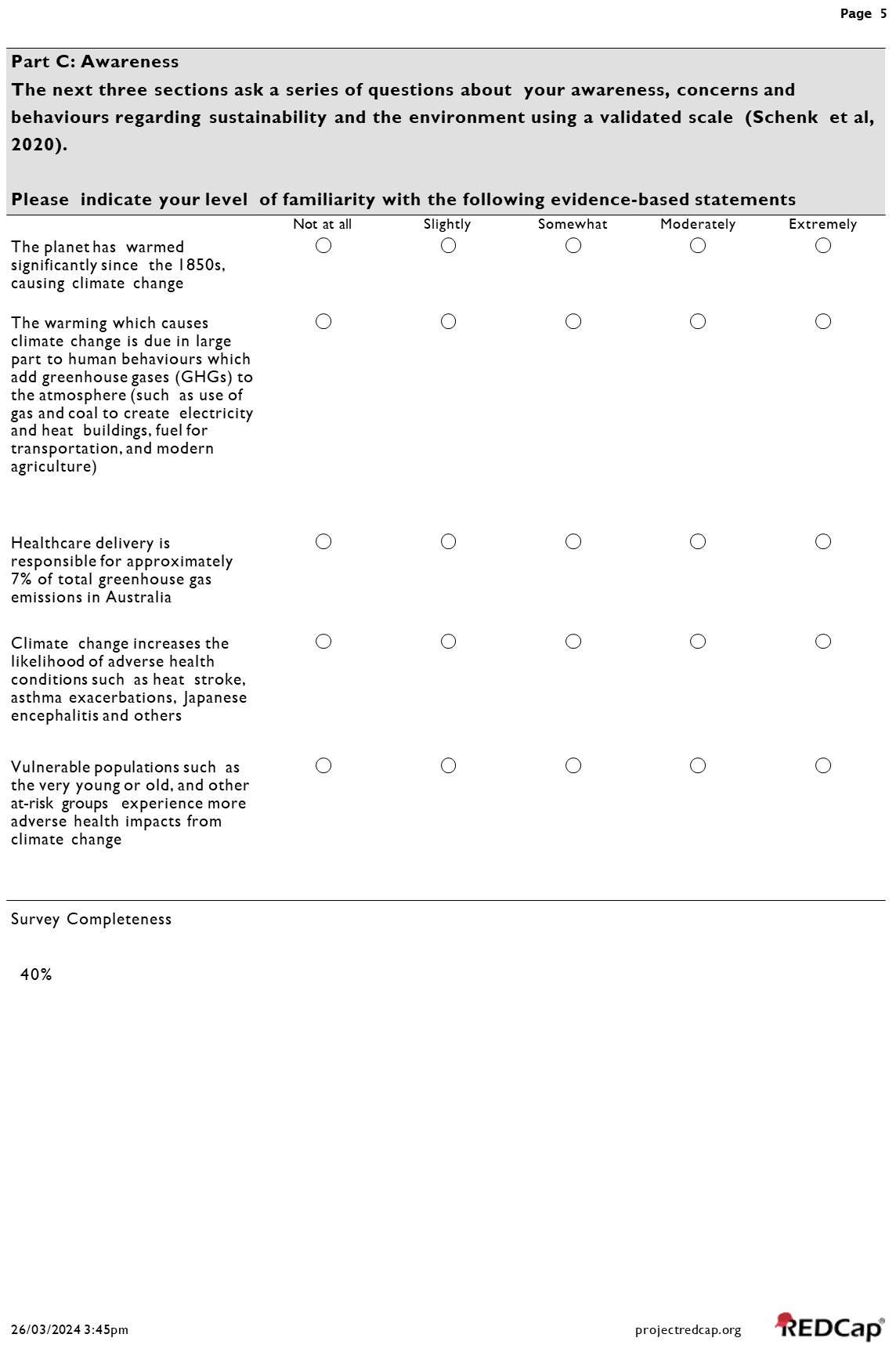
**

**
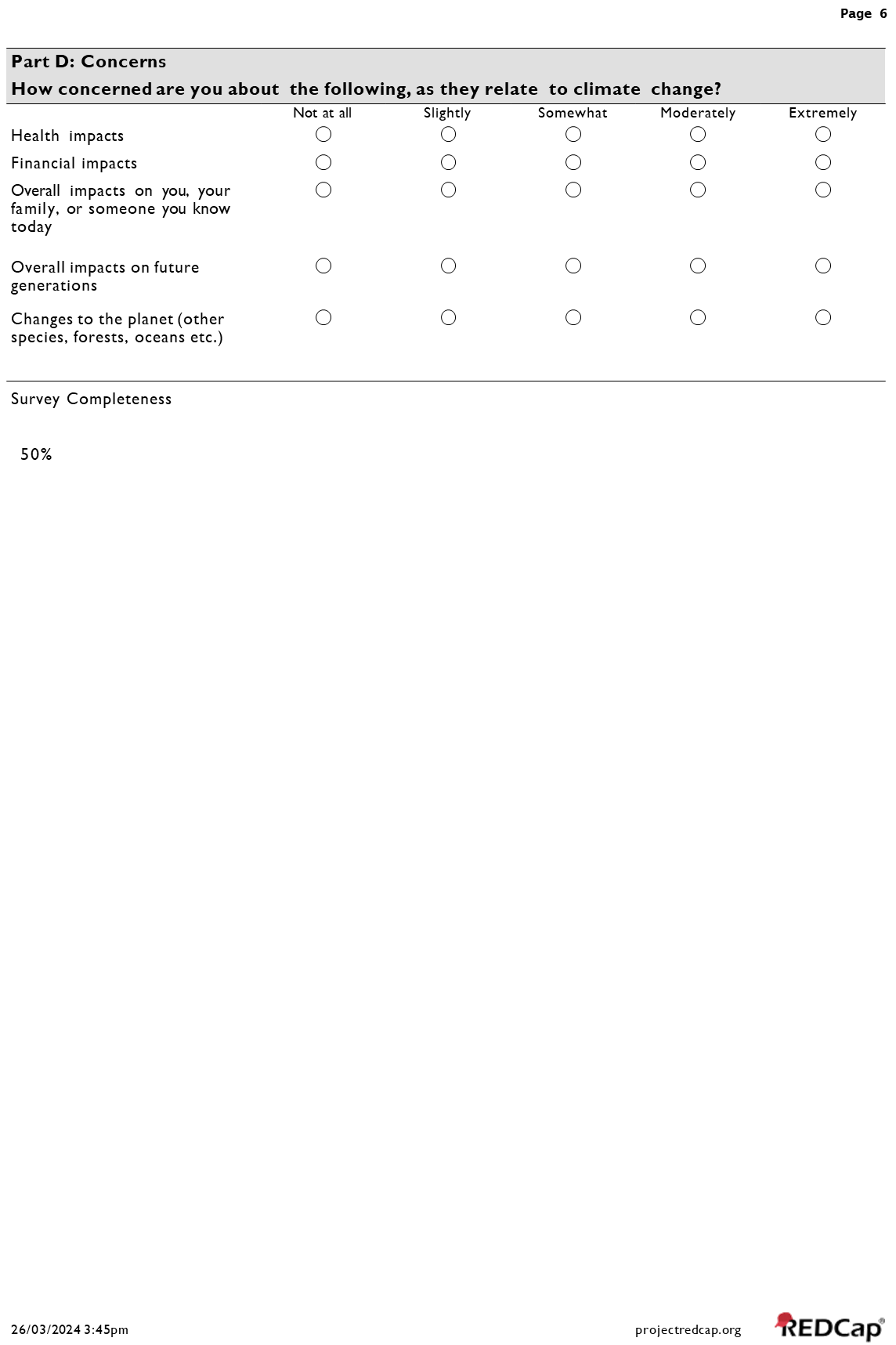
**

**
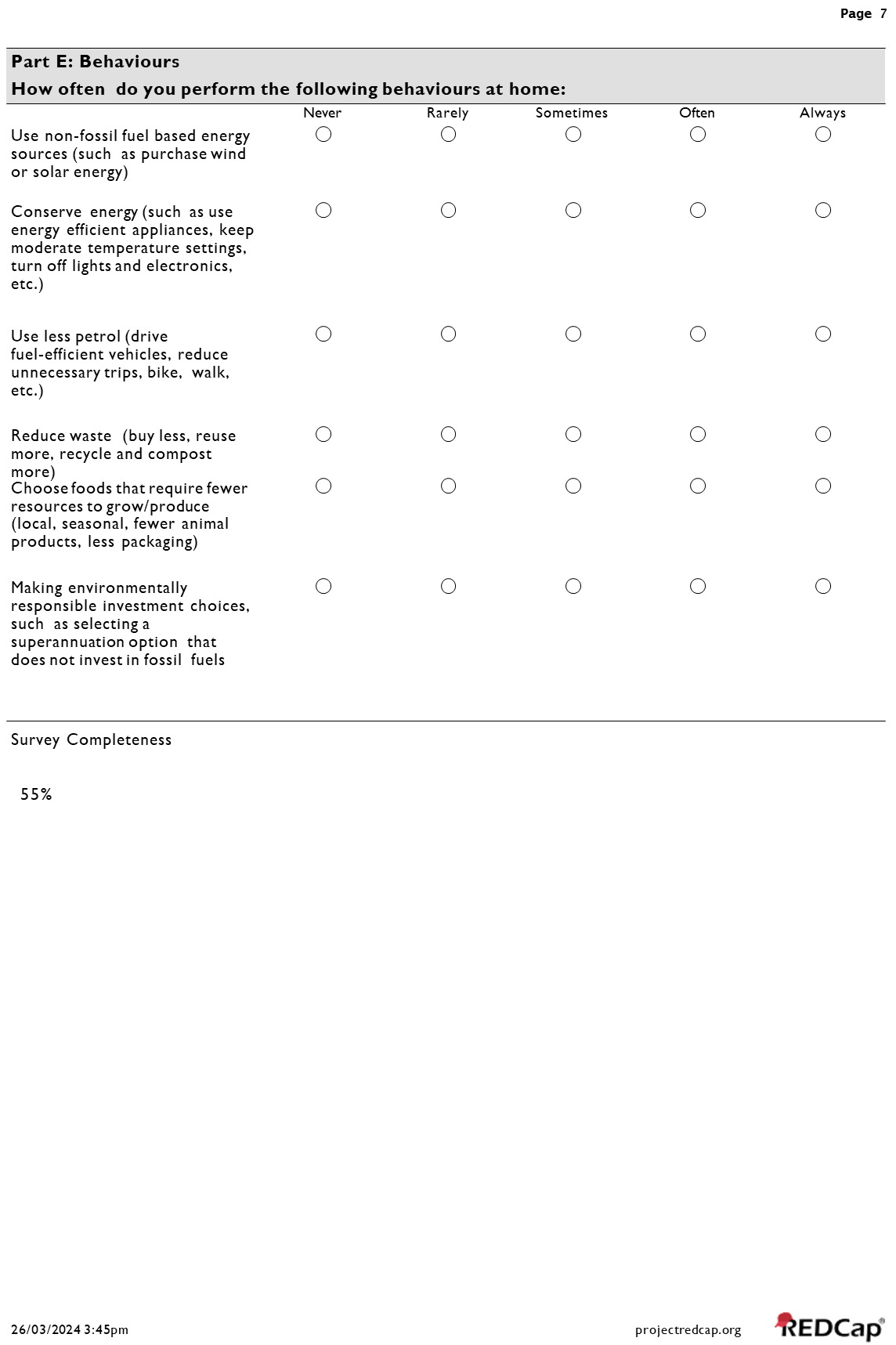
**

**
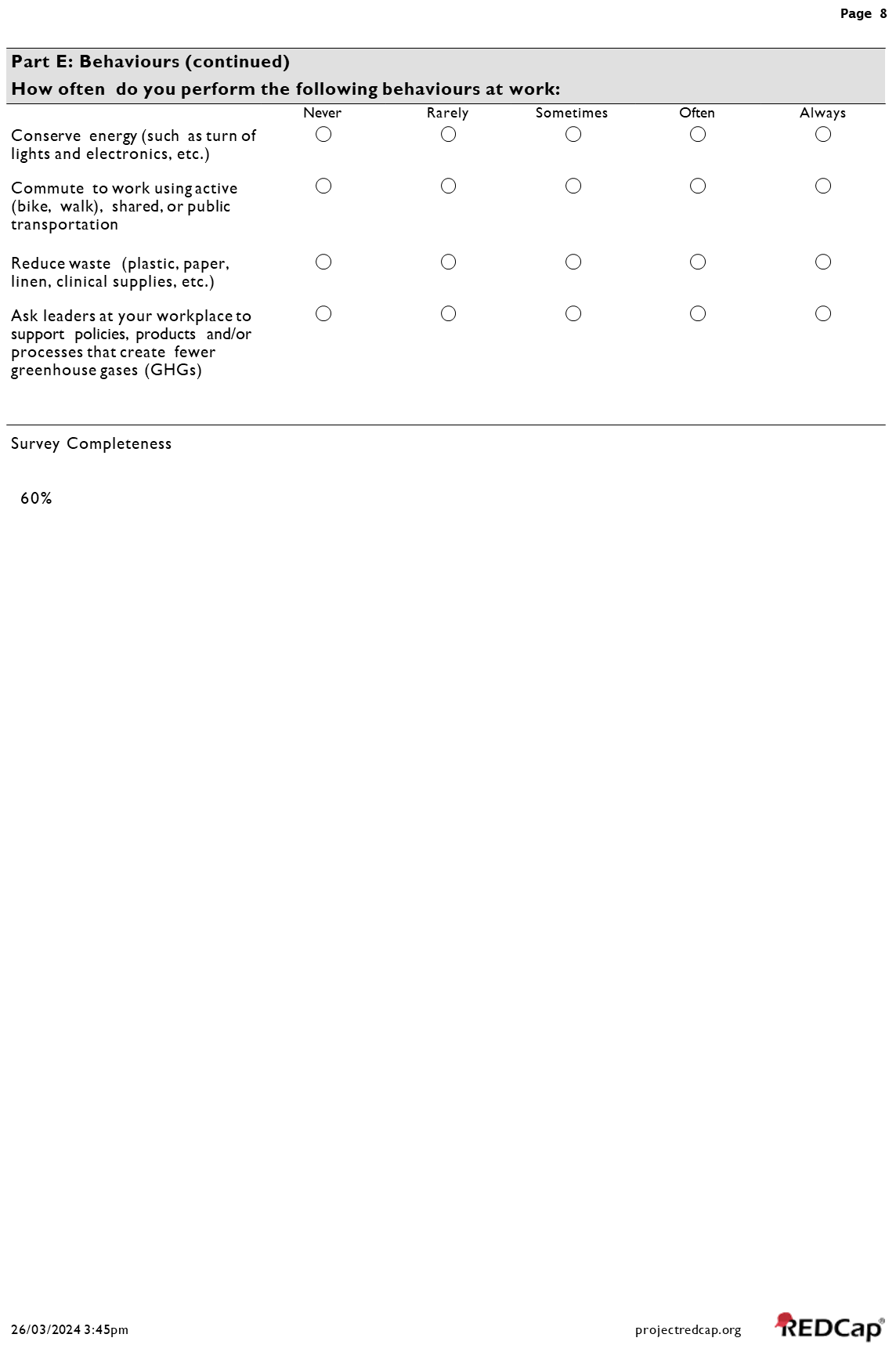
**

**
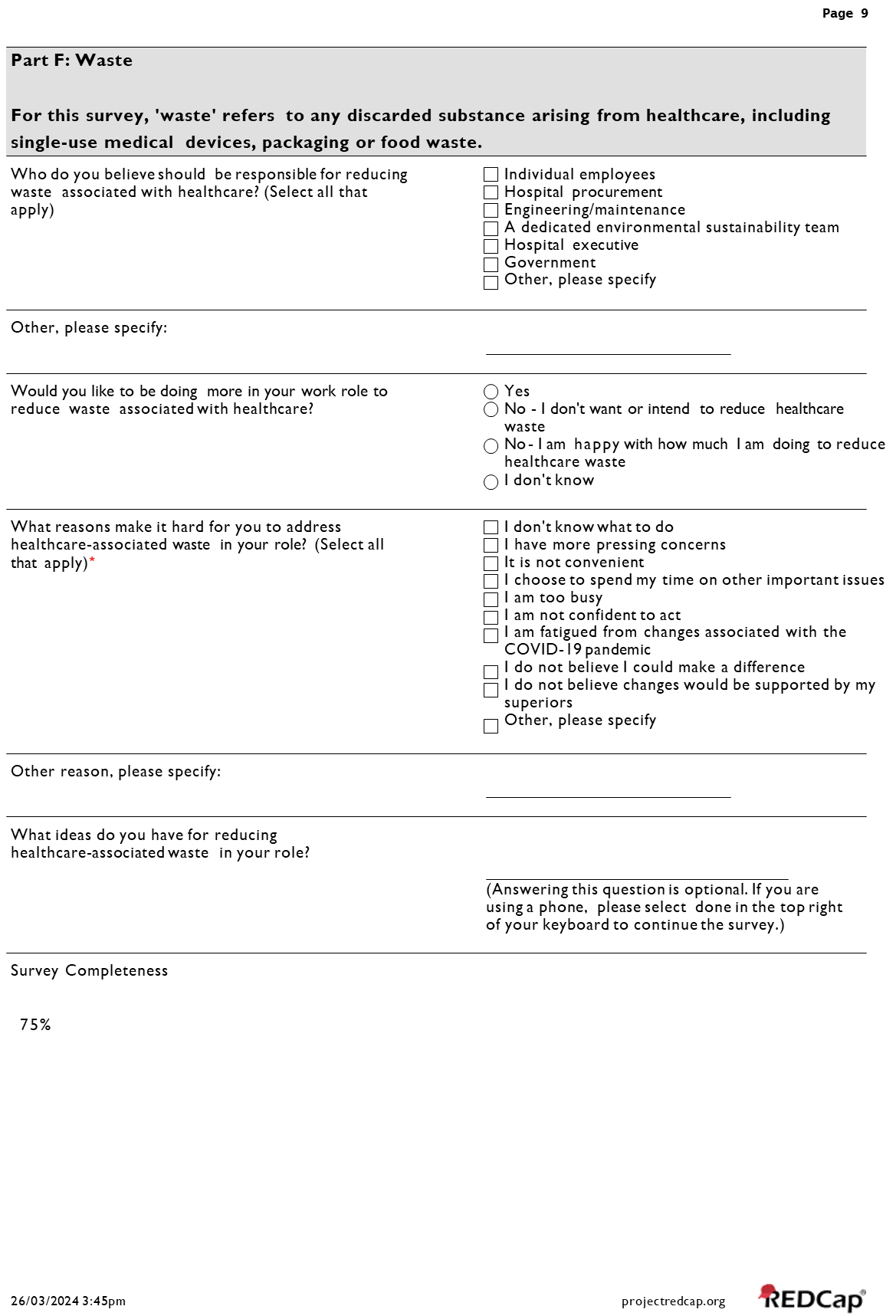
**

**
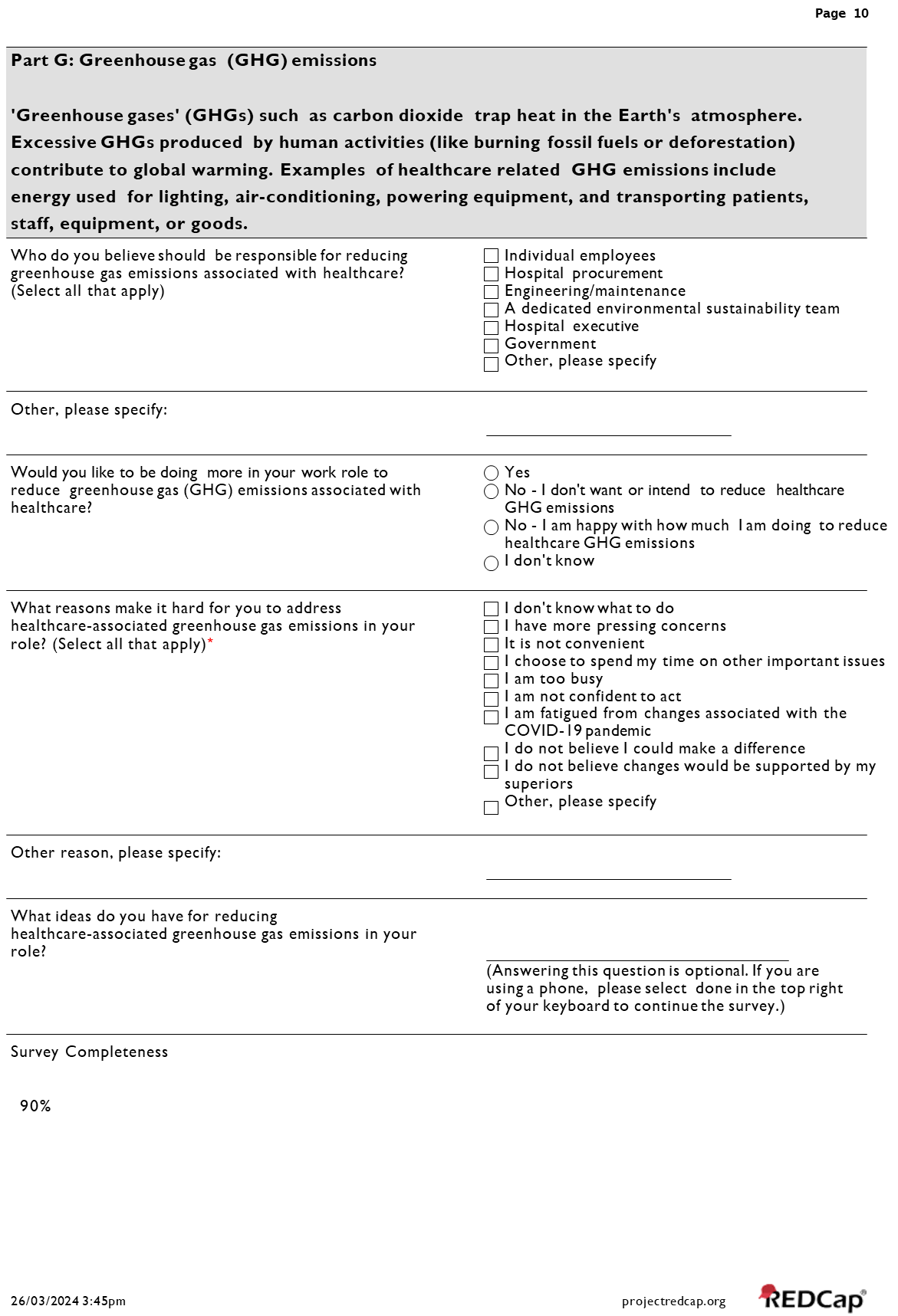
**

**
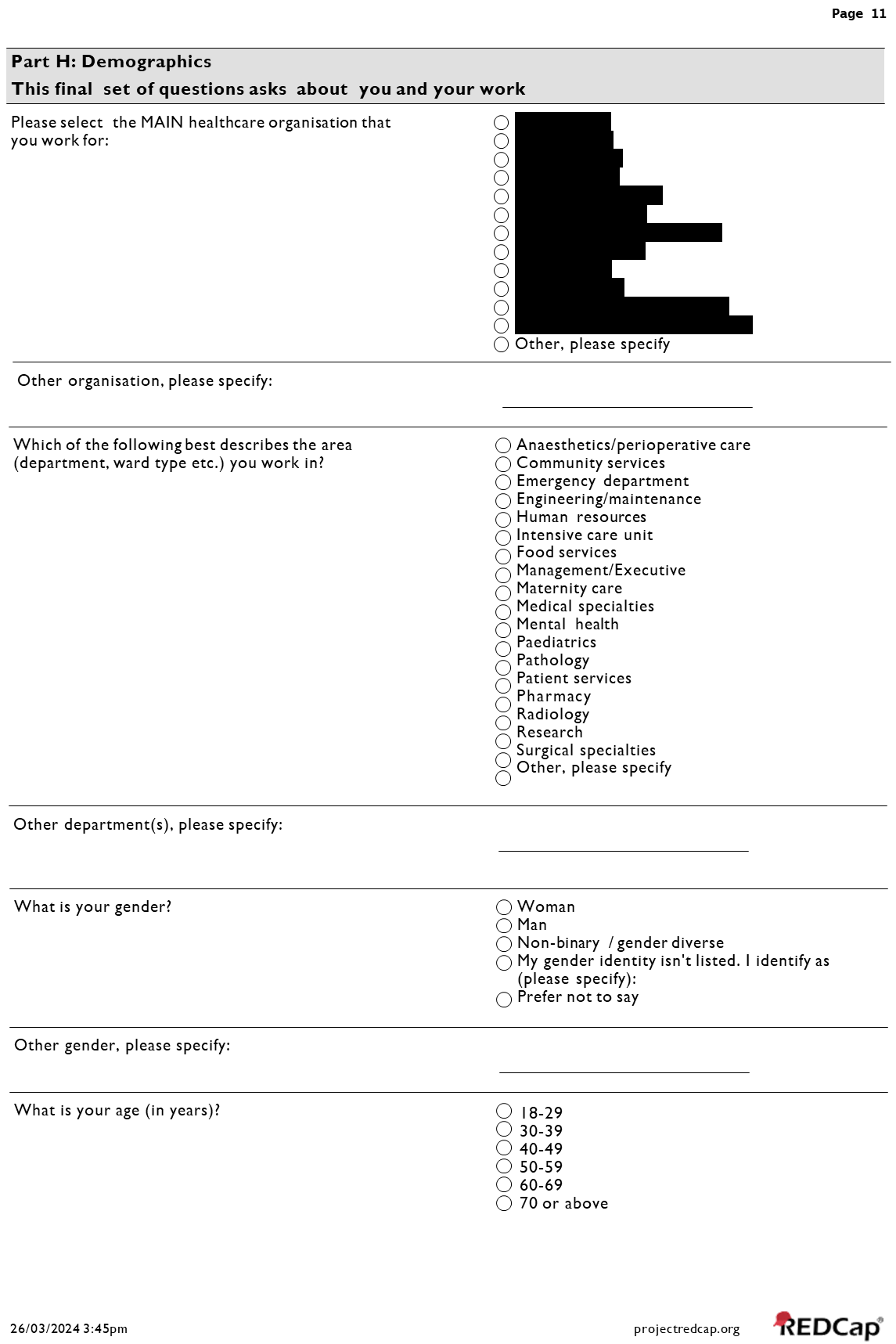
**

**
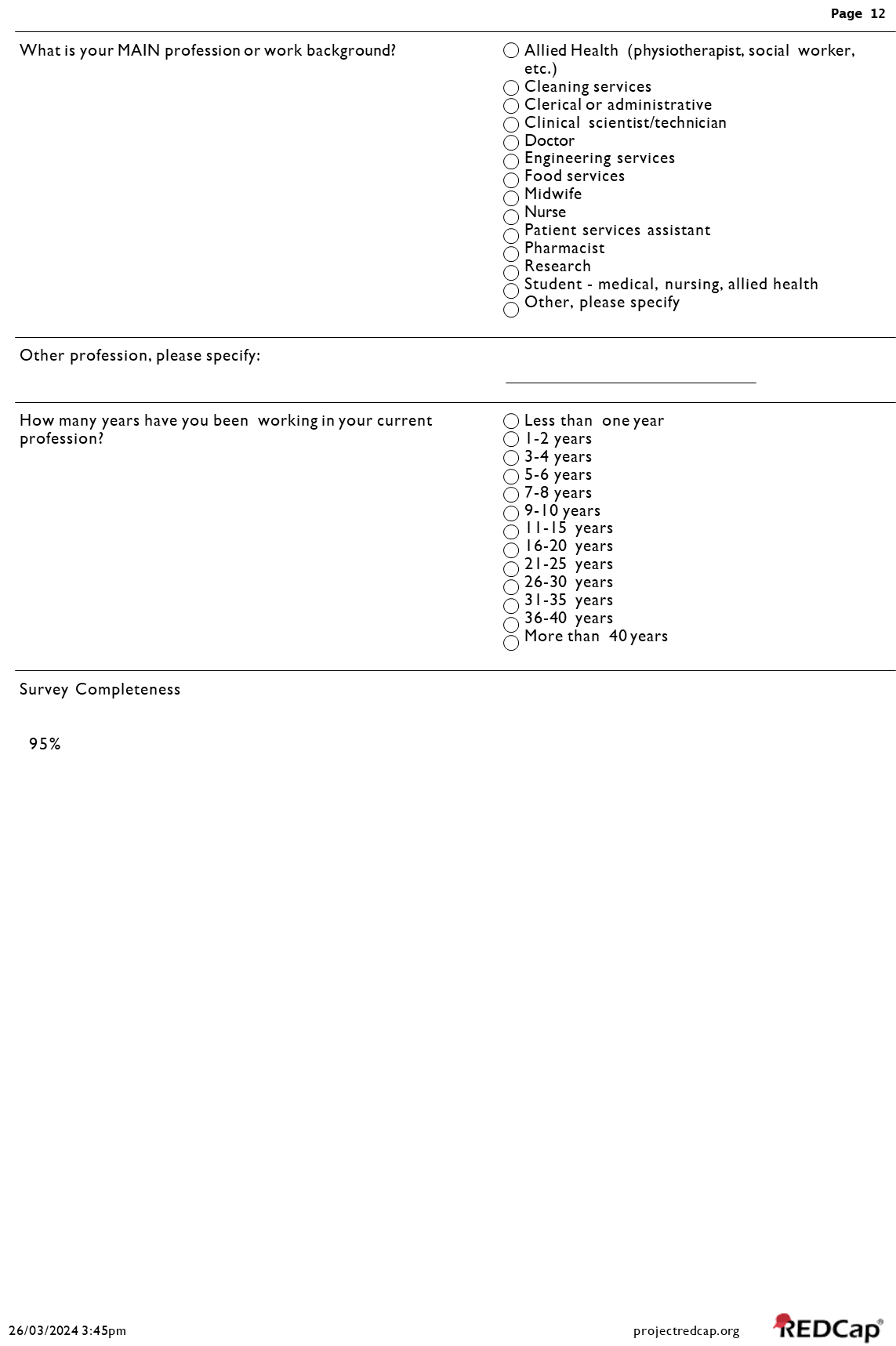
**

**
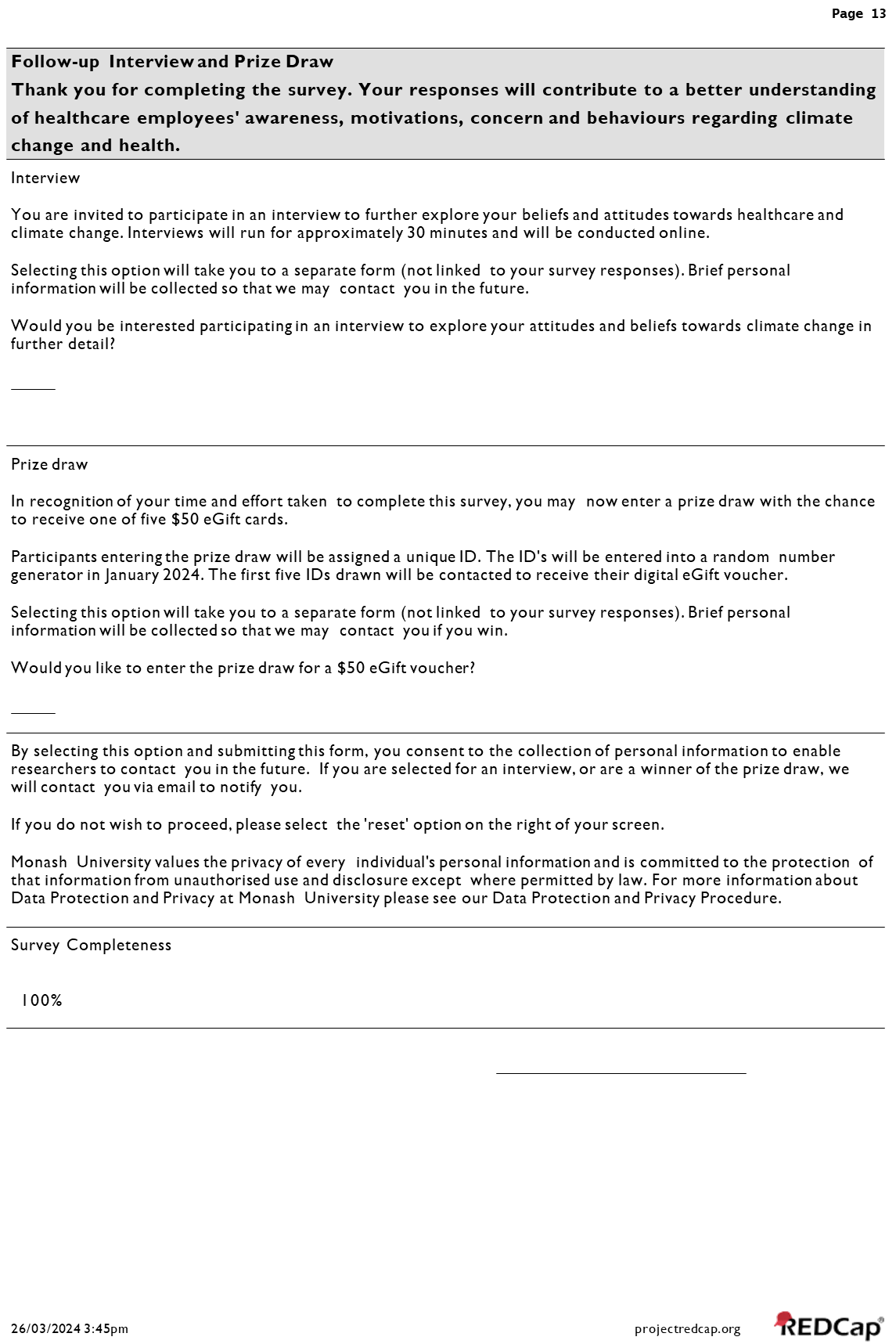
**

# **Appendix File 2: STROBE Statement**

|  | | Item No. | | Recommendation | Page  No. | | Relevant section of manuscript |
| --- | --- | --- | --- | --- | --- | --- | --- |
| **Title and abstract** | | 1 | | (*a*) Indicate the study’s design with a commonly used term in the title or the abstract | 1 | | Title Page |
|  |  |  |  | (*b*) Provide in the abstract an informative and balanced summary of what was done and what was found | 4 | | Abstract |
| Introduction | | | | | | |  |
| Background/rationale | | 2 | | Explain the scientific background and rationale for the investigation being reported | 6 | | Introduction |
| Objectives | | 3 | | State specific objectives, including any prespecified hypotheses | 7 | | Introduction |
| Methods | | | | | | |  |
| Study design | | 4 | | Present key elements of study design early in the paper | 7 | | Methods – Study design |
| Setting | | 5 | | Describe the setting, locations, and relevant dates, including periods of recruitment, exposure, follow-up, and data collection | 7-8 | | Methods - Participants |
| Participants | | 6 | | Give the eligibility criteria, and the sources and methods of selection of participants | 8 | | Methods - Participants |
| Variables | | 7 | | Clearly define all outcomes, exposures, predictors, potential confounders, and effect modifiers. Give diagnostic criteria, if applicable | 8-9 | | Methods – Procedures & Statistical Analysis |
| Data sources/ measurement | | 8 | | For each variable of interest, give sources of data and details of methods of assessment (measurement). Describe comparability of assessment methods if there is more than one group | 8-9 | | Methods – Procedures & Statistical Analysis |
| Bias | | 9 | | Describe any efforts to address potential sources of bias | 9 | | Methods – Statistical Analysis |
| Study size | | 10 | | Explain how the study size was arrived at | 7, 9 | | Methods – Participants & Results |
| Quantitative variables | 11 | | Explain how quantitative variables were handled in the analyses. If applicable, describe which groupings were chosen and why | | 8-9 | Methods – Statistical Analysis | |
| Statistical methods | 12 | | (*a*) Describe all statistical methods, including those used to control for confounding | | 8-9 | Methods – Statistical Analysis | |
|  |  |  | (*b*) Describe any methods used to examine subgroups and interactions | | 8-9 | Methods – Statistical Analysis | |
|  |  |  | (*c*) Explain how missing data were addressed | | 9 | Methods – Statistical Analysis | |
|  |  |  | (*d*) If applicable, describe analytical methods taking account of sampling strategy | | 8-9 | Methods – Statistical Analysis | |
|  |  |  | (*e*) Describe any sensitivity analyses | | N/a | N/a | |
| Results | | | | | | | |
| Participants | 13 | | (a) Report numbers of individuals at each stage of study—eg numbers potentially eligible, examined for eligibility, confirmed eligible, included in the study, completing follow-up, and analysed | | 9 | Results | |
|  |  |  | (b) Give reasons for non-participation at each stage | | 9 | Results | |
|  |  |  | (c) Consider use of a flow diagram | | N/a | N/a | |
| Descriptive data | 14 | | (a) Give characteristics of study participants (eg demographic, clinical, social) and information on exposures and potential confounders | | 9, Table 1 | Results | |
|  |  |  | (b) Indicate number of participants with missing data for each variable of interest | | Table 1 | Results | |
| Outcome data | 15 | | *Cross-sectional study—*Report numbers of outcome events or summary measures | | 10-11 | Results | |
| Main results | 16 | | (*a*) Give unadjusted estimates and, if applicable, confounder-adjusted estimates and their precision (eg, 95% confidence interval). Make clear which confounders were adjusted for and why they were included | | 10-11 | Results | |
|  |  |  | (*b*) Report category boundaries when continuous variables were categorized | | 10-11 | Results | |
|  |  |  | (*c*) If relevant, consider translating estimates of relative risk into absolute risk for a meaningful time period | | N/a | N/a | |

Continued on next page

| Other analyses | 17 | Report other analyses done—eg analyses of subgroups and interactions, and sensitivity analyses | 10, 11, Table 2, Figure 1 | Results |
| --- | --- | --- | --- | --- |
| Discussion | | | | |
| Key results | 18 | Summarise key results with reference to study objectives | 12 | Discussion |
| Limitations | 19 | Discuss limitations of the study, taking into account sources of potential bias or imprecision. Discuss both direction and magnitude of any potential bias | 15 | Discussion |
| Interpretation | 20 | Give a cautious overall interpretation of results considering objectives, limitations, multiplicity of analyses, results from similar studies, and other relevant evidence | 12-16 | Discussion, Conclusion |
| Generalisability | 21 | Discuss the generalisability (external validity) of the study results | 15 | Discussion |
| Other information | |  | | |
| Funding | 22 | Give the source of funding and the role of the funders for the present study and, if applicable, for the original study on which the present article is based | 15 | Funding |

**Note:** An Explanation and Elaboration article discusses each checklist item and gives methodological background and published examples of transparent reporting. The STROBE checklist is best used in conjunction with this article (freely available on the Web sites of PLoS Medicine at http://www.plosmedicine.org/, Annals of Internal Medicine at http://www.annals.org/, and Epidemiology at http://www.epidem.com/). Information on the STROBE Initiative is available at www.strobe-statement.org.

# **Appendix Table 1: Proportion of respondents who prioritised institutional action on climate change above other workplace issues, by gender, age group, occupation, work experience, sector, and location**

Respondents were asked: *How important is it to you that your healthcare organisation prioritise each of the following issues?*

| Variables | Total  N (%) | Climate change rated  lower than personal average  N (%) | Climate change rated  above personal average  N (%) | p-value | Prioritising Climate  N (%) * |
| --- | --- | --- | --- | --- | --- |
| Overall Cohort | 2040 (100.0) | 929 (45.5) | 1111 (54.5) |  | 1635 (80.1) |
| Gender |  |  |  | **<0.001** |  |
| Woman | 1559 (76.4) | 657 (70.7) | 902 (81.2) |  | 1,295 (79.2) |
| Man | 388 (19.0) | 219 (23.6) | 169 (15.2) |  | 280 (17.1) |
| Other | 93 (4.6) | 53 (5.7) | 40 (3.6) |  | 60 (3.7) |
| Age |  |  |  | **0.018** |  |
| 18_29 | 245 (12.0) | 126 (13.6) | 119 (10.7) |  | 188 (11.5) |
| 30_39 | 559 (27.4) | 267 (28.7) | 292 (26.3) |  | 435 (26.6) |
| 40_49 | 486 (23.8) | 222 (23.9) | 264 (23.8) |  | 397 (24.3) |
| 50_59 | 496 (24.3) | 213 (22.9) | 283 (25.5) |  | 403 (24.6) |
| 60+ | 254 (12.5) | 101 (10.9) | 153 (13.8) |  | 212 (13.0) |
| Occupation |  |  |  | **<0.001** |  |
| Doctors | 331 (16.2) | 154 (16.6) | 177 (15.9) |  | 334 (20.4) |
| Nurses/midwives | 611 (30.0) | 247 (26.6) | 364 (32.8) |  | 257 (15.7) |
| Allied Health | 436 (21.4) | 207 (22.3) | 229 (20.6) |  | 272 (16.6) |
| Clerical/administrative | 323 (15.8) | 158 (17.0) | 165 (14.9) |  | 505 (30.9) |
| Other | 339 (16.6) | 163 (17.5) | 176 (15.8) |  | 267 (16.3) |
| Work Experience |  |  |  | **0.05** |  |
| 10+yrs | 1141 (55.9) | 498 (53.6) | 643 (57.9) |  | 713 (43.6) |
| <10yrs | 899 (44.1) | 431 (46.4) | 468 (42.1) |  | 922 (56.4) |
| Sector |  |  |  | 0.420 |  |
| Public | 1937 (95.0) | 881 (94.8) | 1056 (95.1) |  | 1556 (95.2) |
| Private | 102 (5.0) | 48 (5.2) | 54 (4.9) |  | 78 (4.8) |
| Location |  |  |  | 0.176 |  |
| Metropolitan | 1787 (87.6) | 795 (85.6) | 992 (89.4) |  | 1,436 (87.8) |
| Regional/rural | 252 (12.4) | 134 (14.4) | 118 (10.6) |  | 199 (12.2) |

* *Proportions of those ‘Prioritising Climate’ correspond with respondents who rated action on climate change as moderately or extremely important*

# **Appendix Table 2: Average scores for different domains of the Climate and Health Tool***

Refer to Appendix File 1: Main Survey Parts C-E for question stems

| Characteristics | Awareness  Mean (SD) | Concern  Mean (SD) | Behaviours at work  Mean (SD) | Behaviours at home  Mean (SD) |
| --- | --- | --- | --- | --- |
| Overall | 3.09 (0.79) | 3.29 (0.71) | 2.09 (0.79) | 2.48 (0.69) |
| Gender  Female  Male  Other  p-value | 3.11 (0.76)  3.10 (0.81)  2.70 (1.05)  **0.027** | 3.37 (0.62)  3.05 (0.87)  2.96 (1.07)  **<0.001** | 2.07 (0.79)  2.17 (0.78)  2.10 (0.91)  0.243 | 2.49 (0.67)  2.45 (0.72)  2.40 (0.78)  0.616 |
| Age  18-29  30-39  40-49  50-59  60+  p-value | 3.03 (0.74)  3.10 (0.80)  3.11 (0.76)  3.10 (0.81)  3.05 (0.85)  0.378 | 3.26 (0.68)  3.29 (0.70)  3.27 (0.71)  3.31 (0.71)  3.28 (0.78)  0.728 | 1.91 (0.83)  2.03 (0.81)  2.10 (0.78)  2.12 (0.77)  2.29 (0.74)  **<0.001** | 2.28 (0.62)  2.41 (0.68)  2.48 (0.71)  2.56 (0.69)  2.63 (0.67)  **0.005** |
| Profession  Allied Health  Clerical/Admin  Doctor  Nurse/midwife  Other  p-value | 2.98 (0.80)  2.87 (0.82)  3.39 (0.62)  3.14 (0.76)  3.02 (0.86)  **0.001** | 3.29 (0.68)  3.23 (0.77)  3.25 (0.69)  3.36 (0.65)  3.24 (0.82)  **0.015** | 1.99 (0.76)  2.09 (0.80  2.10 (0.74)  2.12 (0.81)  2.14 (0.84)  0.145 | 2.43 (0.64)  2.36 (0.74)  2.69 (0.65)  2.50 (0.66)  2.40 (0.74)  **0.001** |
| Geographical location  Metropolitan  Regional  p-value | 3.10 (0.78)  2.98 (0.86)  0.198 | 3.30 (0.70)  3.17 (0.78)  **0.015** | 2.11 (0.79)  1.93 (0.79)  0.087 | 2.48 (0.68)  2.43 (0.73)  0.561 |
| Sector  Public  Private  p-value | 3.09 (0.79)  3.05 (0.84)  0.503 | 3.29 (0.72)  3.29 (0.68)  0.961 | 2.09 (0.79)  2.02 (0.84)  0.285 | 2.48 (0.69)  2.48 (0.69)  0.916 |
| Work experience  <= 10 years  > 10 years  p-value | 3.05 (0.80)  3.12 (0.78)  0.255 | 3.26 (0.74)  3.31 (0.69)  0.139 | 2.04 (0.84)  2.13 (0.76)  **0.006** | 2.39 (0.70)  2.55 (0.67)  **<0.001** |

**Climate and Health Tool domains (Awareness, Concern, Behaviours at work, and Behaviours at home) are rated on a 5-point scale, scored between 0 and 4*

# **Appendix Table 3: Paired comparison of scores for awareness about climate change, awareness of the health impacts of climate, and awareness of healthcare contribution to climate change, for the entire cohort**

Refer to Appendix File 1: Main Survey Part C for question stems

| Categories according to focus of the question | Mean | SD | Median | Percentile 25 | Percentile 75 |
| --- | --- | --- | --- | --- | --- |
| #1 - Awareness that climate change is happening | 3.40 | 0.77 | 3.50 | 3.00 | 4.00 |
| #2 - Awareness of the health impacts of climate change | 3.15 | 0.99 | 3.50 | 2.50 | 4.00 |
| #3 - Awareness of the healthcare sector’s contribution to climate change | 2.32 | 1.28 | 2.00 | 2.00 | 3.00 |

Category #1 represents the average score to questions 1 and 2 from the Awareness domain

Category #2 represents the average score to questions 4 and 5 from the Awareness domain

Category #3 represents the average score to question 3 from the Awareness domain

Wilcoxon signed-rank test results comparing scores from within the CHANT Awareness domain:

- Comparing Category #1 versus Category #2: p-value <0.01
- Comparing Category #1 versus Category #3: p-value <0.01
- Comparing Category #2 versus Category #3: p-value <0.01

# **Appendix Table 4: Mean score for Climate Change Attitudes Survey (CCAS) questions by gender, age group, profession, work experience, sector, and geographical location**

Refer to Appendix File 1: Main Survey Part B for question stems

| Variables | CCAS  Mean (SD) | p-value |
| --- | --- | --- |
| Overall | 4.36 (0.59) |  |
|  |  |  |
| Gender |  | **<0.001** |
| Woman | 4.41 (0.54) |  |
| Man | 4.24 (0.69) |  |
| Other | 4.03 (0.77) |  |
| Age |  | **0.035** |
| 18_29 | 4.31 (0.54) |  |
| 30_39 | 4.38 (0.55) |  |
| 40_49 | 4.36 (0.60) |  |
| 50_59 | 4.37 (0.61) |  |
| 60+ | 4.31 (0.67) |  |
| Profession |  | **<0.001** |
| Doctors | 4.44 (0.54) |  |
| Nurses/midwives | 4.38 (0.57) |  |
| Allied Health | 4.40 (0.56) |  |
| Clerical/administrative | 4.26 (0.62) |  |
| Other | 4.28 (0.67) |  |
| Work experience |  | **0.05** |
| 10+yrs | 4.38 (0.60) |  |
| <10yrs | 4.33 (0.58) |  |
| Sector |  | 0.758 |
| Public | 4.36 (0.59) |  |
| Private | 4.35 (0.61) |  |
| Geographical location |  | **0.042** |
| Metropolitan | 4.37 (0.59) |  |
| Regional/rural | 4.27 (0.61) |  |

** Climate Change Attitudes Survey responses are rated on a 5-point scale, scored between 1 and 5*

# **Appendix Table 5: Pairwise comparison of responses selecting not knowing what to do about waste versus greenhouse gas (GHG) emissions, for the entire cohort**

From a set of ten options, respondents were asked to select: *What reasons make it hard for you to address healthcare-associated waste in your role?* And; *What reasons make it hard for you to address healthcare-associated greenhouse gas emissions in your role?* Respondents could select more than one answer. Proportions presented represent responses when the option, ‘I don’t know what to do’, was selected (Yes) and not selected (No) in response to each question.

| I don’t know what to do | GHG - No | GHG - Yes | Total |
| --- | --- | --- | --- |
| Waste - No | 850 (41.6) | 395 (19.4) | 1,245 (61.0) |
| Waste - Yes | 106 (5.2) | 689 (33.8) | 795 (39.0) |
| Total | 956 (46.9) | 1,084 (53.1) | 2,040 (100.0) |

**The paired odds ratio is 0.27, exact 95% CI (0.21 – 0.33), McNemar test P<0.001.**

# **Appendix Table 6: Variance in average frequency of reported barriers for waste or greenhouse gas (GHG) emissions by gender, profession, work experience and geographical location**

Respondents were asked: *What reasons make it hard for you to address healthcare-associated waste in your role?* And; *What reasons make it hard for you to address healthcare-associated greenhouse gas emissions in your role?*

|  | Barriers - Waste | | Barriers - GHG | | Barriers – waste or GHG | |
| --- | --- | --- | --- | --- | --- | --- |
| Background variables | **Geometric mean (95% CI)** | **P-value** | **Geometric mean (95% CI)** | **P-value** | **Geometric mean (95% CI)** | **P-value** |
| Gender |  | **0.008** |  | **0.042** |  | **0.012** |
| Woman | 1.84 (1.78 - 1.90) | Reference | 1.58 (1.53 - 1.63) | Reference | 1.67 (1.62 - 1.72) | Reference |
| Man | 1.93 (1.80 - 2.06) | **0.004** | 1.77 (1.65 - 1.90) | **0.019** | 1.72 (1.61 - 1.85) | **0.006** |
| Other | 2.06 (1.76 - 2.40) | **0.005** | 1.76 (1.50 - 2.06) | **0.031** | 1.75 (1.48 - 2.06) | **0.009** |
| Profession |  | **<0.001** |  | **<0.001** |  | **<0.001** |
| Nurses/midwives | 1.82 (1.73 - 1.91) | Reference | 1.62 (1.54 - 1.71) | Reference | 1.69 (1.61 - 1.78) | Reference |
| Doctors | 2.21 (2.06 - 2.37) | **<0.001** | 1.85 (1.72 - 1.98) | **0.027** | 2.02 (1.89 - 2.15) | **0.001** |
| Allied Health | 1.94 (1.83 - 2.05) | **0.035** | 1.59 (1.50 - 1.68) | 0.704 | 1.70 (1.61 - 1.79) | 0.306 |
| Clerical/administrative | 1.55 (1.44 - 1.65) | **<0.001** | 1.37 (1.28 - 1.46) | **<0.001** | 1.37 (1.28 - 1.46) | **<0.001** |
| Other | 1.79 (1.66 - 1.93) | **0.011** | 1.66 (1.54 - 1.78) | 0.100 | 1.62 (1.51 - 1.74) | **0.049** |
| Work experience |  | **0.037** |  | 0.064 |  | **0.032** |
| 10+yrs | 1.81 (1.74 - 1.87) | Reference | 1.58 (1.53 - 1.64) | Reference | 1.64 (1.58 - 1.70) | Reference |
| <10yrs | 1.93 (1.85 - 2.01) | **0.037** | 1.66 (1.59 - 1.73) | 0.064 | 1.73 (1.66 - 1.80) | **0.032** |
| Geographical location |  | 0.278 |  | 0.234 |  | 0.258 |
| Metropolitan | 1.85 (1.80 - 1.91) | Reference | 1.60 (1.56 - 1.65) | Reference | 1.67 (1.62 - 1.72) | Reference |
| Regional/rural | 1.90 (1.75 - 2.06) | 0.278 | 1.72 (1.58 - 1.88) | 0.234 | 1.74 (1.61 - 1.89) | 0.258 |

CI: Confidence interval

Geometric mean and 95% CI were computed for the original variables (not transformed ones)

# Using a linear regression model after log transformation on outcomes, and with the organization clustering effect was considered utilizing cluster robust standard error.

The p-values listed in the same row as the variable name represent an overall p-value comparing all categories simultaneously.

# **Appendix Table 7: Comparison of reported barriers for waste and greenhouse gas (GHG) emissions, by profession**

Respondents were asked: *What reasons make it hard for you to address healthcare-associated waste in your role?* And; *What reasons make it hard for you to address healthcare-associated greenhouse gas emissions in your role?*

|  | Waste | | | | | | GHG | | | | | |
| --- | --- | --- | --- | --- | --- | --- | --- | --- | --- | --- | --- | --- |
| Barriers | **Doctors (n=331)**  **N (%)** | **Nurses (n=611)**  **N (%)** | **Allied Health (n=436)**  **N (%)** | **Administrative (n=323)**  **N (%)** | **Other (n=339)**  **N (%)** | **p-value#** | **Doctors (n=331)**  **N (%)** | **Nurses (n=611)**  **N (%)** | **Allied Health (n=436)**  **N (%)** | **Administrative (n=323)**  **N (%)** | **Other (n=339)**  **N (%)** | **P-value#** |
| I don’t know what to do | 152 (45.9) | 202 (33.1) | 197 (45.2) | 123 (38.1) | 121 (35.7) | **<0.001** | 169 (51.1) | 324 (53.0) | 272 (62.4) | 157 (48.6) | 162 (47.8) | **<0.001** |
| More pressing concerns | 103 (31.1) | 97 (15.9) | 96 (22.0) | 35 (10.8) | 52 (15.3) | **<0.001** | 68 (20.5) | 73 (12.0) | 55 (12.6) | 25 (7.7) | 47 (13.9) | **<0.001** |
| Inconvenient | 86 (26.0) | 124 (20.3) | 77 (17.7) | 19 (5.9) | 47 (13.9) | **<0.001** | 57 (17.2) | 86 (14.1) | 34 (7.8) | 14 (4.3) | 34 (10.0) | **<0.001** |
| Choose to spend time on other issues | 45 (13.6) | 42 (6.9) | 30 (6.9) | 13 (4.0) | 34 (10.0) | **<0.001** | 30 (9.1) | 41 (6.7) | 23 (5.3) | 8 (2.5) | 26 (7.7) | **<0.001** |
| Too busy | 118 (35.7) | 170 (27.8) | 121 (27.8) | 46 (14.2) | 76 (22.4) | **<0.001** | 95 (28.7) | 141 (23.1) | 88 (20.2) | 34 (10.5) | 57 (16.8) | **<0.001** |
| Not confident to act | 58 (17.5) | 95 (15.6) | 87 (20.0) | 54 (16.7) | 63 (18.6) | 0.152 | 48 (14.5) | 90 (14.7) | 69 (15.8) | 42 (13.0) | 62 (18.3) | 0.257 |
| COVID-19 Fatigue | 39 (11.8) | 86 (14.1) | 62 (14.2) | 19 (5.9) | 39 (11.5) | **<0.001** | 25 (7.6) | 63 (10.3) | 31 (7.1) | 11 (3.4) | 25 (7.4) | **<0.001** |
| I don’t believe I could make a difference | 43 (13.0) | 53 (8.7) | 53 (12.2) | 29 (9.0) | 25 (7.4) | **0.043** | 41 (12.4) | 51 (8.4) | 53 (12.2) | 24 (7.4) | 26 (7.7) | **<0.001** |
| Don’t believe changes would be supported | 93 (28.1) | 194 (31.8) | 101 (23.2) | 65 (20.1) | 72 (21.2) | **<0.001** | 77 (23.3) | 134 (21.9) | 72 (16.5) | 46 (14.2) | 50 (14.8) | **<0.001** |
| Other | 69 (20.9) | 132 (21.6) | 87 (20.0) | 48 (14.9) | 69 (20.4) | **<0.001** | 40 (12.1) | 52 (8.5) | 26 (6.0) | 30 (9.3) | 46 (13.6) | **0.006** |

# An overall P-value using a logistic regression model with the organization clustering effect was considered utilizing cluster robust standard error.

# **Appendix Table 8: Thematic analysis of 1157 responses to “What ideas do you have for reducing healthcare-associated waste in your role?”, including illustrative quotes**

| **Theme** | **Subtheme** | **Illustrative Quotes** |
| --- | --- | --- |
| **Improved processes and systems for waste disposal** | **Safe disposal of hazardous / medical waste** | “It causes me enormous concern and 'grief' that there is no strategy for responsible disposal of elastomeric antibiotic infusers used in home healthcare.” (Female, Nurse/Midwife)  “Identify what existing waste can be recycled safely…what items are not yet recycled but could be safely…and put in infrastructure to hold waste until it can be recycled” (Male, Clerical/Administrative) |
|  | **Better access to (general) recycling bins** | “At the bare minimum, having recycling bins.” (Female, Other)  “We need recycle bins…There is one bin and everything goes in it.” (Female, Clerical / Administrative) |
|  | **Improved food waste disposal** | “Donating food not used to local shelters.” (Female, Clerical/Administrative)  “Start a composting initiative. Currently all food waste (from patients and employees) goes into landfill. This could all be composted and used for good.” (Male, Doctor) |
|  | **Perception that recycling efforts are not followed through** | “The staff in my team already separate out the recyclables but they end up getting thrown out to landfill.” (Female, Nurse/Midwife)  “All waste goes into the same skip bin at the end of the day. There is no real recycling program.” (Female, Allied health) |
| **Encouraging a sustainable culture of reduce/reuse/recycle** | **Better education and awareness** | “Education is key.” (Male, Nurse/Midwife)  “Better education for staff included in induction accreditation items. Environment and health are linked and there is a massive disconnect.” (Female, Allied Health)  “Most fellow healthcare workers I speak to are motivated…but don't know what steps to take” (Female, Doctor) |
|  | **More environmentally responsible decision-making** | “Thoughtful prescribing, considering best option for individual but also environment” (Female, Doctor)  “Wastage caused by ordering of tests never read or looked at. There is both a financial cost and also the wastage of consumables to provide the tests.” (Male, Clerical/Administrative)  “Encourage pharmaceutical manufacturers to package responsibly and with bio-degradable products.” (Male, Other)  “More open mindedness from infection prevention.” (Female, Nurse/Midwife) |
|  | **Reducing reliance on single-use items** | “Reduce single use instruments and packaging” (Male, Doctor)  “Look at alternatives to single use plastic” (Female, Nurse) |
|  | **More sustainable everyday practices** | “Build recycling into the flow of the working day – normalise this as a way of life.” (Female, Allied Health)  “Print less and try to keep resources stored electronically rather than as hard copies.” (Female, Allied Health)  “Return to using more reusable equipment rather than single-use.” (Female, Doctor) |
| **Organisational environmental leadership** | **Encouraging local sustainability champions** | “Create a sustainability infrastructure headed by the organisation's sustainability team, with local unit teams. This also ensures that interested clinicians have support and maximises the impact across the organisation.” (Female, Doctor)  “Have every ward allocate an environmental champion just like how they do with falls, nutrition, pressure injury etc” (Female, Nurse) |
|  | **Importance of senior leadership** | “Wider hospital governance and support that makes waste reduction standard practice” (Female, Nurse)  “Changes aren't supported by the hospital executive; therefore department management will not address issues. People have tried with poor to no results.” (Female, Allied health) |

***** Responses such as “Don’t know” or “Unsure” were considered N/A and not included in analysis, whereas others such as “I am not sure what works” or “I don’t know enough to suggest anything” were included

# **Appendix Table 9: Thematic analysis of 592 responses to the question “What ideas do you have for reducing healthcare-associated greenhouse gas emissions in your role?”, including illustrative quotes**

| **Theme** | **Subtheme** | **Quotes** |
| --- | --- | --- |
| **Encouraging sustainability practices in workplace culture** | **Increased education, training and awareness** | “Better education of most efficient use of items, eg sensor lights.” (Female, Nurse)  “I don’t equate the waste to GHG emissions in my daily work. It's not tangible unlike waste or plastic use that we can easily see.” (Prefer not to say, Doctor)  “A few years ago, our executive ran a campaign encouraging staff to turn off power when they leave the office. It really made it front of mind for staff behaviour and was effective at raising awareness.” (Female, Allied health) |
|  | **More efficient everyday practices** | “Stop printing unnecessary documents.” (Male, Other)  “More efficient use of heating and cooling.” (Female, Clerical/Administrative)  “Unplugging things and turning things off when not in use.” (Female, Nurse) |
|  | **Normalising the topic** | “Normalising the topic so it's not just an "external cause', but an everyday issue that affects us all.” (Female, Clinical scientist,)  “Culture change to promote sustainable work” (Female, Nurse) |
| **Energy efficient solutions for the workplace** | **More energy efficient infrastructure** | “Installation of solar; grey-water systems for toilets / irrigation of gardens;” (Female, Allied health)  “Create naturally automated heating and cooling using better materials and designs, install low emission lighting on timers or motion sensors” (Non-binary/Gender Diverse, Clinical Scientist/Technician)  “Movement sensors for lighting - so lights switch on and off according to movement detection. Insulation in buildings to reduce need for heating and cooling.” (Female, Other)  “When building a new hospital structure, incorporate all tech and best practice to reduce waste and energy consumption. Provide a green space (garden)” (Male, Nurse/Midwife) |
|  | **Sustainable Transport alternatives** | “Replacing hospital cars with electric cars, continuing to prioritise hybrid fleet cars” (Male, Doctor)  “I would like our work cars to be electric vehicles or at least hybrids.” (Female, Doctor)  “Provide amenities to support bike riders eg showers, changing rooms, covered bike parks etc” (Female, Nurse/Midwife)  “More solar and EV charging stations” (Male, Allied Health) |
|  | **Waste related solutions** | “Reduce the amount of pharmaceutical waste we generate by sorting waste that doesn't strictly meet the definition of pharmaceutical waste.” (Female, Other)  “Buy less single use equipment, request less plastic packaging or get suppliers to take back and reuse packaging” (Female, Nurse/Midwife)  “Put a restriction on printing files” (Male, Nurse/Midwife) |
| **Bridging individual and systemic changes** | **Lack of individual agency** | “There are only very limited opportunities to make change in a large organisation.” (Female, Other)  “I can't do anything personally” (Male, Doctor) |
|  | **Perceived as a sector and system-level issue** | “That is entirely a role for the government to mandate and hospital executive to ensure they meet, not individual employees!” (Female, Nurse)  “As before, this does not acknowledge the massive role of the organisation in contributing to this. To push responsibility onto the individual within the system is absurd.” (Prefer not to say, Doctor) |
|  | **Insufficient Organisational Action** | “Very minimal, mostly superficial changes have been made” (Female, Nurse)  “Allow staff to charge EV passenger vehicles at work. This was recently stopped by our organisation under the guise of "safety" (Male, Other) |

* Responses such as “Don’t know”, “Unsure” or “Refer to previous answer” were considered N/A and not included in analysis, whereas others such as “I am not sure what works” or “I don’t know enough to suggest anything” were included

# **Appendix Figure 1: Respondents expressing a desire to do more to address waste and greenhouse gas emissions in their role**

**
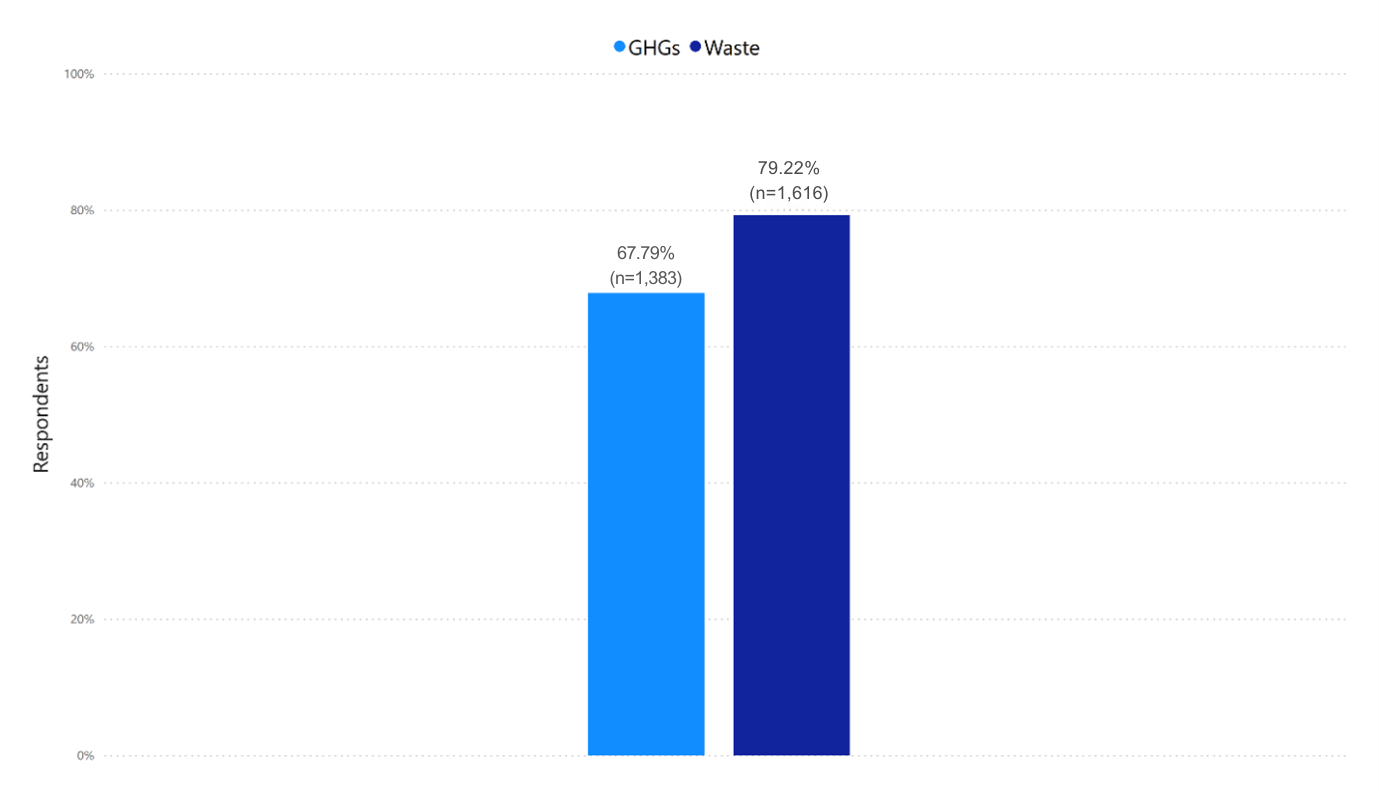
**Respondents were asked: *Would you like to be doing more in your work role to reduce waste associated with healthcare?* And; *Would you like to be doing more in your work role to reduce greenhouse gas emissions associated with healthcare?*

# **Appendix Figure 2: Perceived responsibility for addressing healthcare-associated waste and greenhouse gas emissions**

| Respondents were asked: *Who do you believe should be responsible for reducing waste associated with healthcare?* And; *Who do you believe should be responsible for reducing greenhouse gas emissions associated with healthcare?*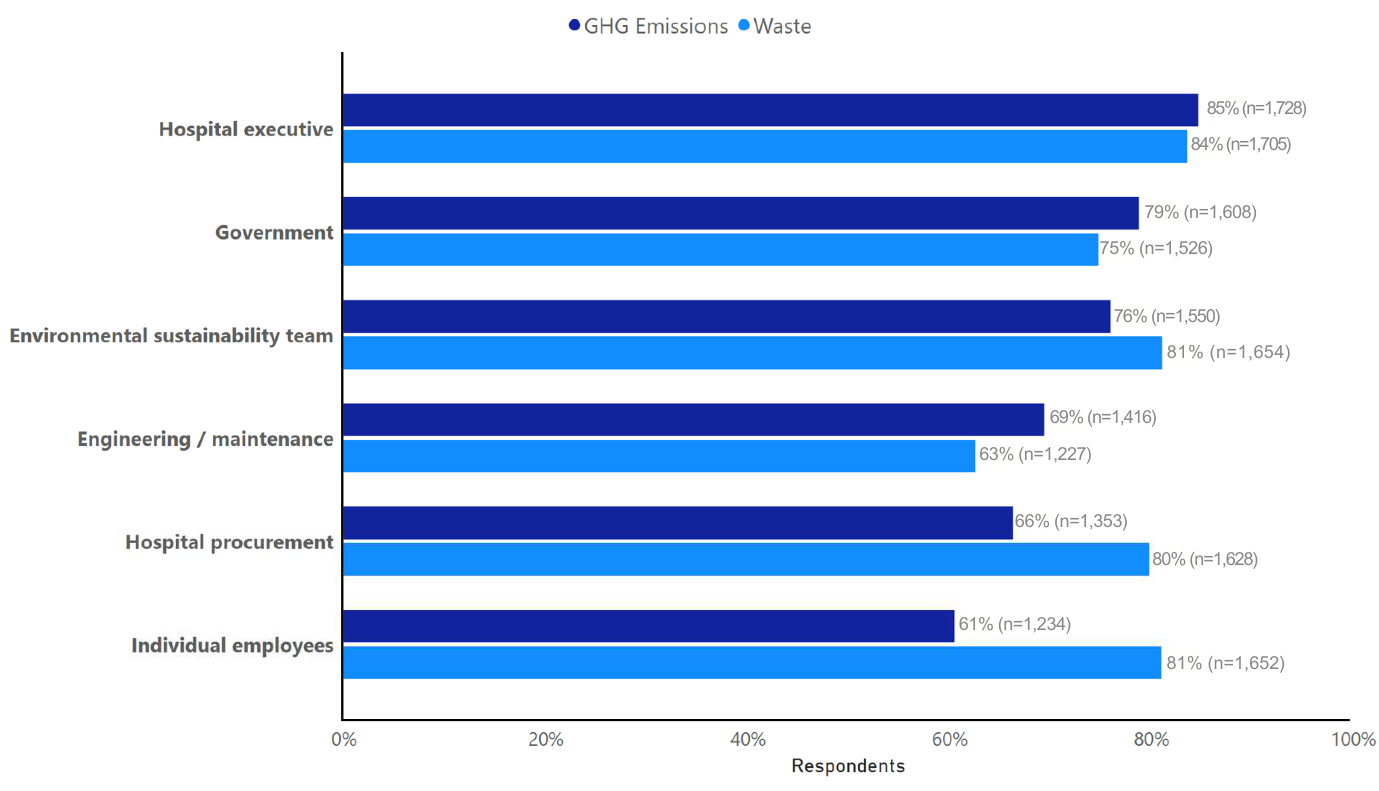 * p-values were calculated using Fisher's exact test adjusted for survey design, with organisational clustering accounted for through survey-weighted methodology. Tabulated figure data, including p-values included below:**Category** | **GHG %** | **Waste %** | **p-value** |
| --- | --- | --- | --- |
| Hospital executive | 85 | 84 | 0.4110 |
| Government | 79 | 75 | 0.0026 |
| Environmental sustainability team | 76 | 81 | <0.001 |
| Engineering / maintenance | 69 | 63 | <0.001 |
| Hospital procurement | 66 | 80 | <0.001 |
| Individual employees | 61 | 81 | <0.001 |
